# Cerebrospinal fluid metabolomic profiles can discriminate patients with leptomeningeal carcinomatosis from patients at high risk for leptomeningeal metastasis

**DOI:** 10.18632/oncotarget.20983

**Published:** 2017-09-18

**Authors:** Byong Chul Yoo, Jun Hwa Lee, Kyung-Hee Kim, Weiwei Lin, Jong Heon Kim, Jong Bae Park, Hyun Jin Park, Sang Hoon Shin, Heon Yoo, Ji Woong Kwon, Ho-Shin Gwak

**Affiliations:** ^1^ Biomarker Branch, Research Institute, National Cancer Center, Goyang, Republic of Korea; ^2^ Department of Cancer Biomedical Science, National Cancer Center, Graduate School of Cancer Science and Policy, Goyang, Republic of Korea; ^3^ Center for Pediatric Cancer, National Cancer Center, Goyang, Republic of Korea; ^4^ Neuro-oncology Clinic, National Cancer Center, Goyang, Republic of Korea

**Keywords:** cerebrospinal fluid, metabolome, leptomeningeal carcinomatosis, profile, diagnosis

## Abstract

**Purpose:**

Early diagnosis of leptomeningeal carcinomatosis (LMC) is necessary to improve outcomes of this formidable disease. However, cerebrospinal fluid (CSF) cytology is frequently false negative. We examined whether CSF metabolome profiles can be used to differentiate patients with LMC from patients having a risk for development of LMC.

**Results:**

A total of 10,905 LMIs were evaluated using PCA-DA. The LMIs defined Group 2 with a sensitivity of 85% and a specificity of 91%. After selecting 33 LMIs, including diacetylspermine and fibrinogen fragments, the CSF metabolomics profile had a sensitivity of 100% and a specificity of 93% for discriminating Group 1b from the other groups. After selecting 21 LMIs, including phosphatidylcholine, the CSF metabolomics profile differentiated LMC (Group 2) patients from the high-risk groups of Group 3 and Group 4 with 100% sensitivity and 100% specificity.

**Materials and Methods:**

We prospectively collected CSF from five groups of patients: Group 1a, systemic cancer; Group 1b, no tumor; Group 2, LMC; Group 3, brain metastasis; Group 4, brain tumor other than brain metastasis. All metabolites in the CSF samples were detected as low-mass ions (LMIs) using mass spectrometry. Principal component analysis-based discriminant analysis (PCA-DA) and two search algorithms were used to select the LMIs that differentiated the patient groups of interest from controls.

**Conclusions:**

Analysis of CSF metabolite profiles could be used to diagnose LMC and exclude patients at high-risk of LMC with a 100% accuracy. We expect a future validation trial to evaluate CSF metabolic profiles supporting CSF cytology.

## INTRODUCTION

Cerebrospinal fluid (CSF) surrounds the central nervous system (CNS). CSF circulates throughout the entire neuraxis and transports neuro-transmitters, bioactive substances such as hormones, and active and passive secretory compounds of brain cells [1]. Researchers analyze CSF to detect evidence or activity, or both, of CNS disease [2, 3]. Some proteins, such as tumor-specific antigens, can be used as diagnostic and quantitative biomarkers for brain tumors (e.g., germ cell tumors) [4]. However, in general, tumor-specific antigen is expressed in only a proportion of patients [5], and total protein is non-specific for monitoring of disease activity [6].

Leptomeningeal carcinomatosis (LMC) is a dismal terminal stage disease of solid cancer that rapidly deteriorates the patient's performance. Overall survival of patients with LMC is approximately 6–8 weeks, without definitive treatment [7]. Thus, early diagnosis is necessary to improve patient outcome from this formidable disease. The low probability of detection of floating cancer cells within a small volume (< 5 ml) of CSF results in the approximately 50–60% sensitivity of a single CSF cytology sample, even when overt LMC-related symptoms are present [8, 9]. Modern neuroimaging of gadolinium-enhanced magnetic resonance imaging (MRI) reveals typical leptomeningeal enhancement [8], but that is neither early event nor pathognomonic for diagnose of LMC with only 60–70% sensitivity [10].

Metabolites can be detected as low-mass ions (LMIs) using mass spectrometry (MS). The resulting metabolomic profiles of secretion and bio-fluids in cancer patients reveal the unique characteristics of the cancer environment. Use of LMIs is expected to be a valid method for early diagnosis of cancer development and metastasis [11]. MS-based metabolomics analysis of CSF has been used to identify signatures of malignant glioma [12, 13]. These pilot studies have been used to examine correlations between metabolite expression and tumor grade or prognosis, but the clinical usefulness of metabolomics analysis of CSF remains unclear.

We used liquid chromatography-tandem mass spectrometry (LC-MS/MS) and analyzed MS-based metabolic profiles of CSF in a large number of patients (*n* = 196). We adopted principal component analysis-based discriminant analysis (PCA-DA) to examine differences in metabolomic profiles between groups of patients. We also set discriminative LMIs through search algorithm II of germination, growth, and shrinkage modules, and evaluated whether the selected LMIs could discriminate LMC patients from patients at high risk for the development of LMC, including brain metastasis or brain tumors.

## RESULTS

### Clinical characteristics of patients

The results for the demographic characteristics of each patient group (i.e., Group 1a, systemic cancer; Group 1b, no tumor; Group 2, LMC; Group 3, brain metastasis; Group 4, brain tumor other than brain metastasis) are presented in Table 1. The group of 196 patients were nearly equally distributed to each gender (male:female = 101:95). We did not restrict age during CSF sampling, so age ranged from 1 to 83 years (median age, 52 years).

**Table 1:** Clinical characteristics of patients (*n* = 196)

Characteristics	Total	1a Cancer control (*n* = 58)	1b Non-tumorous control (*n* = 42)	2 LMC (*n* = 67)	3 Brain metastasis (*n* = 9)	4 Brain tumors (*n* = 20)
Gender						
Male	101 (52)a	42 (72)	18 (43)	26 (39)	5 (56)	10 (50)
Female	95 (48)	16 (28)	24 (57)	41 (61)	4 (44)	10 (50)
Median age	52	19	60	53	57	24
Combined disease		Leukemia (45) Bladder (12) Lymphoma (7) Bone (7) Breast (5) Colorectal (5) Prostate (5) Others (14)	Unruptured An (43) Moyamoya (17) Cbr. a. stenosis (10) BPH (7) Others (23)	NSLCL (64) Breast (21) Stomach (6) Others (9)	NSCLC (44) Others (56)	Glioma (35) MBL (15) GCT (15) Others (35)

Abbreviations: LMC, Leptomeningeal carcinomatosis; An, aneurysm; BPH, benign prostate hyperplasia; Cbr. a., cerebral artery; NSCLC, non-small cell lung cancer; MBL, Medulloblastoma; GCT, germ cell tumor

aNumbers in parenthesis are vertical proportion.

Among Group 1a patients, leukemia was the most common cancer type (45%), followed by bladder cancer (12%), lymphoma, osteosarcoma/chordoma (7% each), breast cancer, colorectal cancer, prostate cancer (5% each), and other cancers. For sampling of neither CNS nor systemic cancer (Group 1b, no tumor), craniotomy for unruptured cerebral aneurysm was the most prevalent procedure (43%), followed by Moyamoya disease (17%), and bypass surgery for cerebral artery stenosis (10%). The most frequent primary cancer affecting Group 2, LMC patients was non-small cell lung cancer (64%), followed by breast cancer (21%), stomach cancer (6%), and other organ cancers (9%, each of small cell lung cancer, melanoma, retinoblastoma, glioblastoma and malignancy of unknown origin). Non-small cell lung cancer was also the most common source of brain metastasis (44%) among Group 3 patients. Gliomas were the most common diagnosis for Group 4 patients with brain tumors (35%), followed by medulloblastoma (15%) and germ cell tumors (15%).

### Candidate LMIs for LOMEs 1 and 2

**Figure 1:**
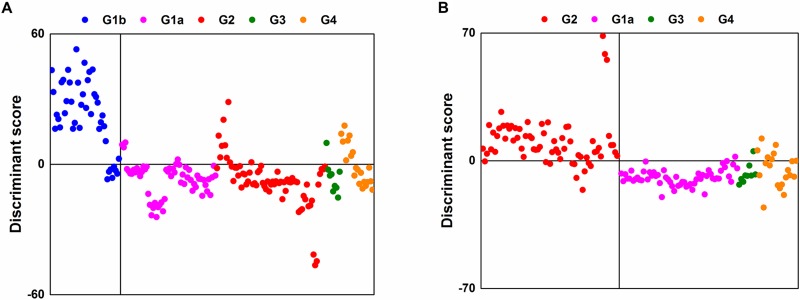
Separation results using PCA-DA with all LMIs (**A**) Group 1b vs. groups of 1a/2/3/4. (**B**) Group 2 vs. groups of 1a/3/4.

A total of 10,905 LMIs were used for the Low-Mass ion discriminant Equations (LOMEs). The results for the discriminants for Group 1b vs. groups of 1a/2/3/4 (sensitivity, 83%; specificity, 85%) and Group 2 vs. groups of 1a/3/4 (sensitivity, 85%; specificity, 91%) using PCA-DA are presented in Figure 1A and 1B, respectively. All LMIs in the logarithmic peak table contributed to the PCA-DA discriminant scores (DSs).

Search algorithm I discovered 314 candidate LMIs for Group 1b vs. groups 1a/2/3/4 and 241 candidate LMIs for Group 2 vs. groups 1a/3/4, respectively. The discriminant results for the former had a sensitivity of 81% and a specificity of 79%; the latter had a sensitivity of 79% and a specificity of 87%. When PCA-DA was performed using the peak table of only candidate LMIs, the sensitivity and specificity values for Group 1b vs. groups 1a/2/3/4 were 81% and 83%, respectively; the values for Group 2 vs. groups 1a/3/4 were 87% and 91%, respectively. A comparison of the results for the 10,905 LMIs and the selected LMIs revealed that the remarkable reduction in the number of LMIs did not cause a sharp decline in separation performance.

### LOME 1-33 for discriminating cancer patients from patients without cancer, and LOME 2-21 for discriminating LMC patients from patients with brain metastasis or brain tumors

**Figure 2:**
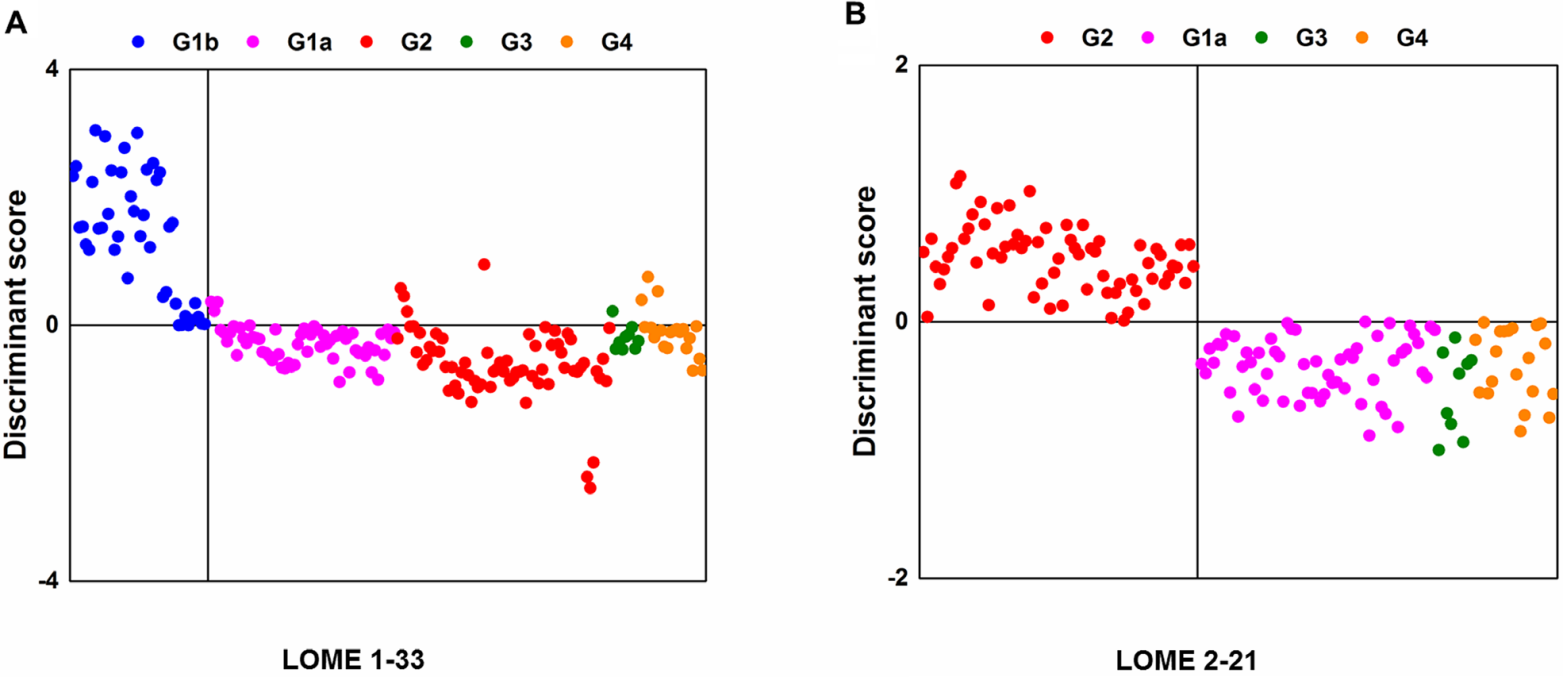
Separation results with discriminative LMIs (**A**) Group 1b vs. groups of 1a/2/3/4 (LOME 1-33). (**B**) Group 2 vs. groups of 1a/3/4 (LOME 2-21).

Search algorithm II individually sought out 33 LMIs (LOME 1-33, Figure 2A, Supplementarty Table 1) for discriminating Group 1b from a set of groups 1a/2/3/4 and 21 LMIs (LOME 2-21, Figure 2B, Table 2) for discriminating Group 2 from a set of groups 1a/3/4 as discriminative LMIs. The results for the separations for the former (sensitivity, 100%; specificity, 93%) and the latter (sensitivity, 100.0%; specificity, 100.0%) by using each set of discriminative LMIs are presented in Figure 2A and 2B. The results for Group 2 as a target of screening indicated that the sensitivity was 94.03% (4 false negatives for LOME 1-33) and specificity was 100.0%, based on a combination of LOME 1-33 (negative DS) and LOME 2-21 (positive DS), although this result was for the training set.

**Table 2:** Twenty-one low-mass ions discriminating patients with leptomeningeal metastasis (Group 2) from other groups of patients at high risk for developing leptomeningeal metastasis (groups of 1a/3/4)

Selected LMI (^a^*m/z*)	^b^Compound	Metabolite Name	Adduct	Adduct MW (Da)	Compound MW (Da)	Delta
100.0753	HMDB11749	2-Piperidinone	M+H	100.07569	99.06841392	0.00039
	HMDB40518	2,5-Dihydro-2,4-dimethyloxazole	M+H	100.07569	99.06841392	0.00039
	HMDB34587	1-Pyrrolidinecarboxaldehyde	M+H	100.07569	99.06841392	0.00039
	HMDB60309	(2R)-2-Hydroxy-2-methylbutanenitrile	M+H	100.07569	99.06841392	0.00039
192.1597	n.a.					
195.0887	HMDB01847	Caffeine	M+H	195.087652	194.0803756	0.001048
	HMDB14962	Enprofylline	M+H	195.087652	194.0803756	0.001048
205.0076	n.a.					
207.1478	HMDB41445	Agrocybenine	M+H	207.149189	206.1419132	0.001389
	HMDB60656	Monoethylglycinexylidide	M+H	207.149189	206.1419132	0.001389
248.1654	HMDB14597	Meperidine	M+H	248.164505	247.1572289	0.000895
	HMDB41913	Ketobemidone	M+H	248.164505	247.1572289	0.000895
270.9564	n.a.					
275.2792	n.a.					
349.0596	n.a.					
401.0730	n.a.					
458.0316	n.a.					
477.0209	n.a.					
491.3350	n.a.					
645.5672	n.a.					
663.4520	n.a.					
780.5522	HMDB07890	PC(14:0/22:5(4Z,7Z,10Z,13Z,16Z))	M+H	780.553781	779.546505	0.001581
	HMDB08689	PC(22:5(7Z,10Z,13Z,16Z,19Z)/14:0)	M+H	780.553781	779.546505	0.001581
	HMDB08140	PC(18:2(9Z,12Z)/18:3(6Z,9Z,12Z))	M+H	780.553781	779.546505	0.001581
	HMDB08108	PC(18:1(9Z)/18:4(6Z,9Z,12Z,15Z))	M+H	780.553781	779.546505	0.001581
	HMDB08075	PC(18:1(11Z)/18:4(6Z,9Z,12Z,15Z))	M+H	780.553781	779.546505	0.001581
	HMDB08016	PC(16:1(9Z)/20:4(8Z,11Z,14Z,17Z))	M+H	780.553781	779.546505	0.001581
	HMDB08015	PC(16:1(9Z)/20:4(5Z,8Z,11Z,14Z))	M+H	780.553781	779.546505	0.001581
	HMDB07984	PC(16:0/20:5(5Z,8Z,11Z,14Z,17Z))	M+H	780.553781	779.546505	0.001581
	HMDB07922	PC(14:1(9Z)/22:4(7Z,10Z,13Z,16Z))	M+H	780.553781	779.546505	0.001581
	HMDB07891	PC(14:0/22:5(7Z,10Z,13Z,16Z,19Z))	M+H	780.553781	779.546505	0.001581
	HMDB08141	PC(18:2(9Z,12Z)/18:3(9Z,12Z,15Z))	M+H	780.553781	779.546505	0.001581
	HMDB08171	PC(18:3(6Z,9Z,12Z)/18:2(9Z,12Z))	M+H	780.553781	779.546505	0.001581
	HMDB08656	PC(22:5(4Z,7Z,10Z,13Z,16Z)/14:0)	M+H	780.553781	779.546505	0.001581
	HMDB08624	PC(22:4(7Z,10Z,13Z,16Z)/14:1(9Z))	M+H	780.553781	779.546505	0.001581
	HMDB08495	PC(20:5(5Z,8Z,11Z,14Z,17Z)/16:0)	M+H	780.553781	779.546505	0.001581
	HMDB08463	PC(20:4(8Z,11Z,14Z,17Z)/16:1(9Z))	M+H	780.553781	779.546505	0.001581
	HMDB08430	PC(20:4(5Z,8Z,11Z,14Z)/16:1(9Z))	M+H	780.553781	779.546505	0.001581
	HMDB08235	PC(18:4(6Z,9Z,12Z,15Z)/18:1(11Z))	M+H	780.553781	779.546505	0.001581
	HMDB08236	PC(18:4(6Z,9Z,12Z,15Z)/18:1(9Z))	M+H	780.553781	779.546505	0.001581
	HMDB08204	PC(18:3(9Z,12Z,15Z)/18:2(9Z,12Z))	M+H	780.553781	779.546505	0.001581
816.2967	n.a.					
892.3155	n.a.					
1058.4993	n.a.					
1231.3553	n.a.					

a, mass-to-charge ratio; b, information used in searching candidate metabolite in Human Metabolome Database (HMDB, http://www.hmdb.ca). Search condition; Mass tolerance ± 0.005 and H^+^ adduct in positive mode

### Candidate metabolites for LMIs in LOME 1-33

Supplementary Table 1 shows the candidate metabolites of 33 LMIs discriminating cancer patients from patients without cancer. The relative amounts of LMIs in each patient group are also presented (Supplementary Figure 1). Except for 11 LMIs, the metabolic compounds in Supplementary Table 1 matched with the LMI mass information.

**Figure 3:**
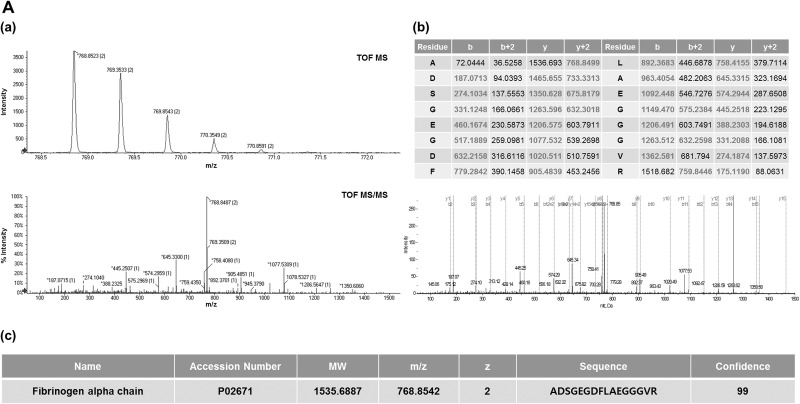

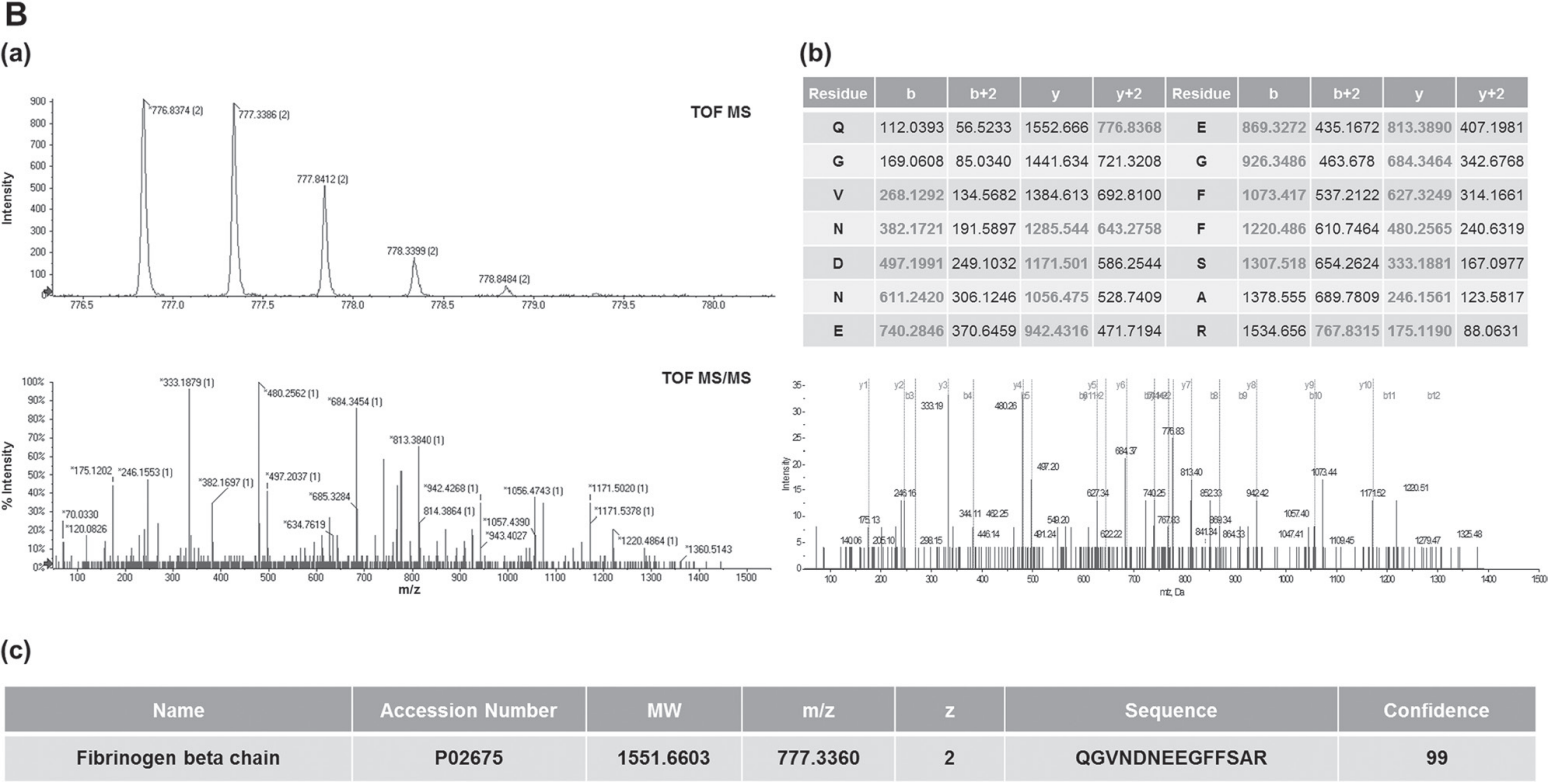
Identification of CSF metabolic peptides in LOME 1-33 MS and MS/MS spectra of CSF metabolites with 768.8542 *m/z* (**A**-**a**) and 777.3360 *m/z* (**B**-**a**). MS and MS/MS pattern analyses were performed using a Triple TOF 5600+ mass spectrometer. Peptide sequence analysis of CSF metabolites with 768.8542 *m/z* (**A**-**b**) and 777.3360 *m/z* (**B**-**b**). Identification of 768.8542 *m/z* (**A**-**c**) and 777.3360 *m/z* (**B**-**c**) as fibrinogen alpha (ADSGEGDFLAEGGGVR) and beta (QGVNDNEEGFFSAR) fragments, respectively.

**Figure 4:**
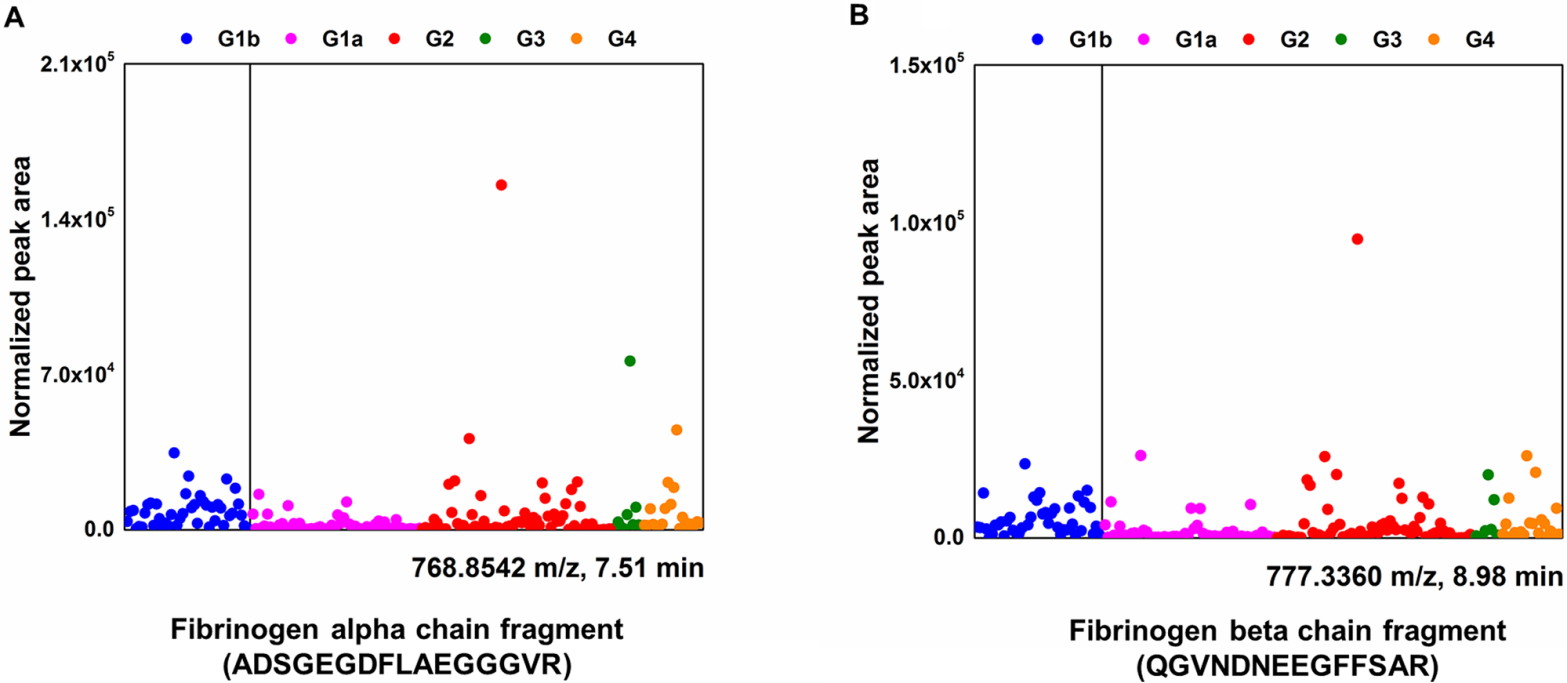

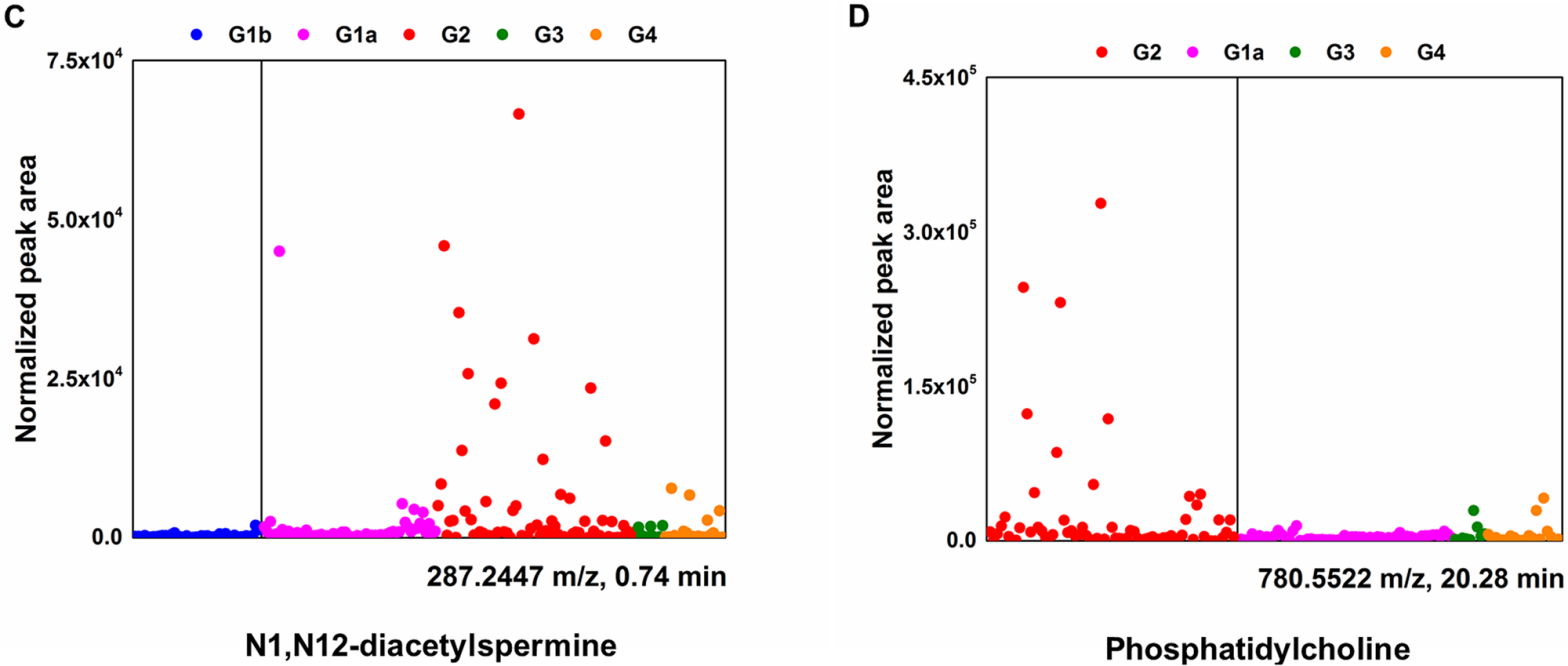
Differential CSF levels of fibrinogen alpha/beta chain, N1, N12-diacetylspermine, and phosphatidylcholine in five different groups Normalized peak area (arbitrary unit) represents relative amounts of identified metabolites in CSF. Fibrinogen alpha/beta chain (**A** and **B**) and N1, N12-diacetylspermine (**C**) are one of the components in LOME 1-33; phosphatidylcholine (**D**) is in LOME 2-21.

Among 31 LMIs, 2 (768.8542 and 777.3360 *m/z*) were peptide fragments; the LC-MS/MS analysis revealed that these peptide fragments were fibrinogen alpha (ADSGEGDFLAEGGGVR) and beta (QGVNDNEEGFFSAR) fragments, respectively (Figure 3A and 3B). Relative amounts of two CSF metabolic peptide fragments were lower in CSF from patients with systemic cancer (Group 1a) compared to other groups (Figure 4A and 4B). A search of the Human Metabolome Database (HMDB, www.hmdb.ca) revealed that an LMI of 248.1654 *m/z* was one of two pain control drugs (meperidine or ketobemidone); this LMI was highly expressed in Group 2 patients (Supplementary Figure 2). Because both drugs have the same molecular formula (C_15_H_21_NO_2_), they were denoted as the same metabolite. Three LMIs (134.0957, 219.1205, and 229.1310 *m/z*) were found at a relatively high prevalence in Group 2 and Group 4 patients (Supplementary Figure 1). The 134.0957 *m/z* LMI candidate was one of two compounds: 1) tranylcypromine, a monoamine oxidase inhibitor for treatment of major depression, dysthymic disorder, and atypical depression, or 2) tranylcypromine or 1,2,3,4-tetrahydroisoquinoline, which are derivatives formed in the body as metabolites of some drugs [14]. 5-Methoxydimethyltryptamine, a potent serotonergic hallucinogen found in mammalian brains, and N-despropyl ropinirole, a metabolite of ropinirole (a non-ergoline dopamine agonist), were selected as the candidates for a 219.1205 *m/z* LMI. Metyrapol is a metabolite of metyrapone. Metyrapone is a drug used for the diagnosis of adrenal insufficiency and occasionally for treatment of Cushing's syndrome. Metyrapol was a candidate for a 229.1310 *m/z* LMI. Greater amounts of this LMI were found in Group 2 and Group 4 patients, compared with the other groups (Supplementary Figure 1). Two LMIs (333.1436 and 410.1669 or 410.1673 *m/z*) showed relatively higher peaks in Group 1b patients. A 333.1436 *m/z* LMI was a metabolite of flecainide (meta-O-dealkylated flecainide), which is used to treat a variety of cardiac arrhythmias, or of zanamivir, which is used to inhibit neuraminidase. Coenzyme Q is a substrate for cytochrome *bc*1 complex subunit Rieske, mitochondrial; it was selected as the metabolite for the 410.1669 and 410.1673 *m/z* LMIs.

The 287.2447 and 298.0974 *m/z* LMIs were identified as an N1, N12-diacetylspermine, which is a polyamine used as a marker to efficiently detect cancers, and 5’-methylthioadenosine, a naturally occurring sulfur-containing nucleoside present in all mammalian tissues, respectively. The relative amounts of N1, N12-diacetylspermine were up-regulated in CSF from the patients with LMC (Group 2) (Figure 4C).

### Candidate metabolites for LMIs in LOME 2-21

Twenty-one LMIs were selected for discriminating patients with LMC (Group 2) from other groups of patients (Table 2). The HMDB search revealed that 5 of these 21 LMIs corresponded to metabolic compounds in the database. The metabolic compound candidates for the 195.0887 *m/z* LMI were: 1) caffeine, the most widely consumed psychostimulant drug, and 2) enprofylline, which is used for treatment of asthma and chronic obstructive pulmonary disease, and for the management of cerebrovascular insufficiency, sickle cell disease, and diabetic neuropathy. Only 248.1654 *m/z* LMI was selected in both LOME 1-33 and LOME 2-21. It appeared as a pain control drug metabolite of meperidine/ketobemidone and was also highly expressed in Group 2 patients (Supplementary Figure 2).

Many phosphatidylcholine derivatives with slightly different chemical structures were selected as the candidate metabolite for the 780.5520 *m/z* LMI because they were composed of the same chemical elements (Table 2). The amount of phosphatidylcholine was relatively higher in CSF from patients with LMC (Group 2) compared with patients with other types of cancer (groups of 1a, 3, and 4) (Figure 4D).

## DISCUSSION

Our study is the first report of CSF metabolomic profiles that discriminate LMC patients from other high-risk groups of patients, including those with brain tumor and brain metastasis. We set the objectives as early diagnosis and screening of LMC patients and obtained 100% sensitivity.

### Advancement of MS and LOME to identify discriminating LMIs

Dekker et al. performed an experiment that was similar to ours; they discriminated the CSF of metastatic breast cancer between patients with or without LMC [15]. The values for sensitivity and specificity for detecting LMC were 79% and 76%, respectively. This accuracy was not better than current diagnostic methods using CSF cytology or gadolinium-enhanced MRI [10]. Another weakness of their study was the protocol to define patients without LMC. Their results indicated that 4 out of 46 control group patients had a positive or suggestive MRI finding, but they did not repeat CSF cytology for these patients. And MRI findings were not available for 20 patients (43%). Freilich and Straathof found that MRI (+) and CSF cytology (–) patients have a diagnostic probability of 75% [8, 10]. In our study, all Group 2 patients had positive findings of both MRI and CSF cytology. To avoid including patients with latent LMC in the control group, we selected all Group 3 and Group 4 patients to be followed more than 3 months after CSF sampling.

**Figure 5:**
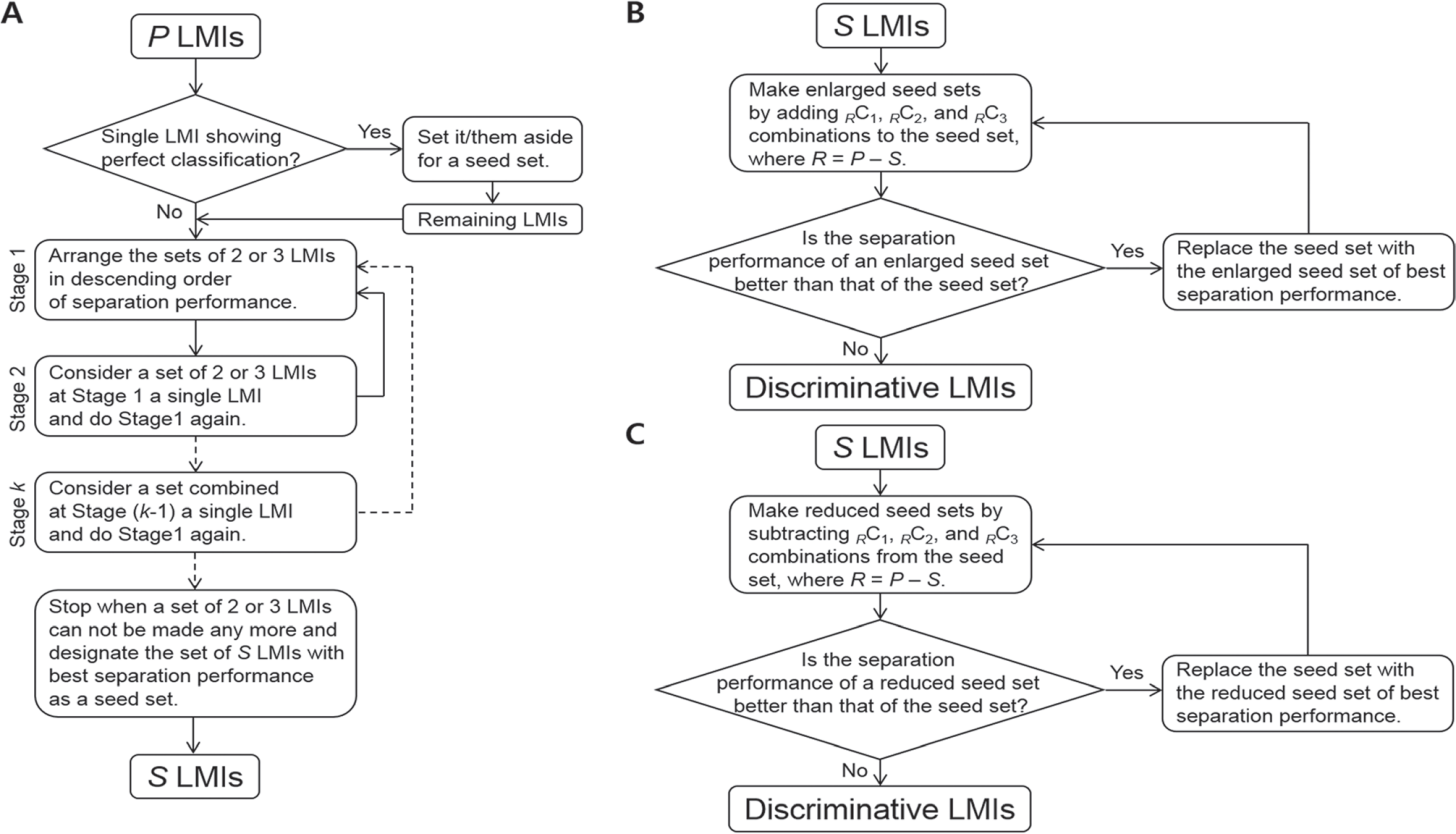
Search algorithm II (**A**) Germination module. (**B**) Growth module. (**C**) Shrinkage module.

Although Dekker et al. used matrix assisted laser desorption ionization-time of flight mass spectrometry (MALDI-TOF MS), they could identify only 164 discernible peaks in year 2005; we found 10,905 identifiable LMIs. This apparently increased number of LMIs might have contributed to the ability to discriminate Group 2 from groups of 1a/3/4 at values of 85% sensitivity and 91% specificity, but the number of LMIs is not necessarily proportional to the accuracy. Unlike our two prior studies [11, 16], we applied the LOME scheme to the triple-TOF MS data in this study, selected the LOMEs (LOME 1-33 and 2-21), and obtained an accuracy of 100% for discriminating LMC from the patients at high risk for LMC. Considering the difference in magnitude of the mass intensity, the magnitude threshold of 0.01 was empirically determined for search algorithm I. Search algorithm II was revised by equipping the shrinkage module to obtain a more compact set of discriminant LMIs (Figure 5). As a result, LOME 1-33 and LOME 2-21 were very accurate at discriminating cancer patients from patients without cancer, and LMC patients from patients with brain metastasis or brain tumors, respectively (Figure 2). Furthermore, the triple-TOF MS data provided information on the actual amounts of each discriminant LMI. This very important difference in approach, compared with that used for our previous MALDI-TOF based metabolic profiling, provided strong support for our present results.

### Identification of LMIs as metabolomes

Five patient groups (i.e., free of CNS cancer or tumor, CNS tumor free with systemic cancer, LMC, brain metastasis, and brain tumor other than brain metastasis) were enrolled in this study (Table 1). Each patient group underwent various therapies based on the disease, and the therapy type affected our metabolomics profiling results. For example, many of the identified metabolites or selected candidate metabolites that were identified as discriminant LMIs appeared as a chemical drug used in the treatment of patients, especially meperidine and ketobemidone (Table 2 and Supplementary Table 1). This result also supported the sensitivity of the discriminant LMI selection from the 10,905 LMIs, and indicated that the CSF metabolomics profiling results were reliable.

N1, N12-diacetylspermine and fibrinogen alpha chain fragments were identified as an LMI in the LOME 1-33 that discriminated cancer patients from patients without cancer. N1, N12-diacetylspermine is an amine metabolic compound known to be a urine tumor biomarker. Increased urine concentrations of N1, N12-diacetylspermine occurs in many types of cancers (e.g., breast cancer [17, 18], colorectal cancer [18], non-small cell lung cancer [19], bladder cancer [20], metastatic and primary brain tumors including grade 3 and 4 gliomas and primary CNS lymphoma [21], hepatocellular carcinoma [22], and leukemia [23]). To the best of our knowledge, our results are the first to suggest that the CSF concentrations of N1, N12-diacetylspermine might be useful for selecting patients with cancers or brain tumors.

Fibrinogen fragment is also associated with inflammation and cancers. Up-regulation of fibrinogen production in blood can be a useful prognostic factor in various types of cancers (e.g., ovarian [24], bladder [25], lung [26], renal [27], and colorectal [11]). Increased CSF fibrinogen levels have not been reported for solid cancer metastases, but deranged levels have been found in CSF from children with leukemia [28]. We identified fibrinogen alpha (ADSGEGDFLAEGGGVR) and beta (QGVNDNEEGFFSAR) fragments in CSF. Differential levels of both metabolic peptides in CSF might be useful for discriminating CNS tumor free patients with systemic cancer (Group 1a) from other groups of patients.

Phosphatidylcholine was one of LMIs in the LOME 2-21 that discriminated LMC patients from patients with brain metastasis or brain tumors. High phosphatidylcholine levels have been consistently observed in tissues from various types of cancers (e.g., breast [29], colorectal [30], and thyroid papillary cancer [31]). Phosphatidylcholine is required for breast cancer cell survival [29]. Dysregulation of oxidized phosphatidylcholine in CSF occurs in patients with childhood acute lymphoblastic leukemia with methotrexate-related neurotoxicity [32]. Phosphatidylcholine derivatives are intermediate products of phosphatidylcholine, which is a major constituent of cell membranes and has a major role in membrane-mediated cell-signaling and transportation [33]. We did not directly measure choline kinase activity in this study, but we could assume that the increased phosphatidylcholine in the CSF of patients with LMC results from increased cell membrane turn-over as cancer cells proliferating in the CSF of patients. Abnormal regulation of phosphatidylcholine in CSF linked to disease has not been reported, but we expect that N1, N12-diacetylspermine and phosphatidylcholine might be a very useful biomarker set for LMC screening.

### Clinical usefulness of CSF metabolomics profiles for LMC

Locasale et al. analyzed metabolites of CSF in 10 patients with malignant gliomas and 7 patients without malignancy [12]. Using a targeted MS-based metabolomics platform, profiling of the relative levels of 124 polar metabolites, and hierarchical clustering of CSF metabolite composition, they found 3 metabolic signatures that can discriminate malignant gliomas from control samples. Although they suggested that it is important to perform serial sampling of CSF in addition to tissue biopsy, they did not provide results to support the clinical usefulness of their targeted MS-based metabolomic profiles in terms of molecular sub-classification of malignant gliomas or a treatment response. For this purpose, re-setting target metabolomes based on new molecular classification of malignant gliomas (i.e., production of 2-hydroxyglutarate from *IDH1* mutation) [34], and subsequent prospective CSF profiling study is necessary. We believe that our CSF metabolomics profiles from patients with LMC reflect the unique environment of floating cancer cells and their driver mutations that adapt them to an aqueous phase. This environment is different from the nutrient and growth factor enriched environment relevant to solid cancer survival. We are analyzing the genomic profiles of CSF from patients with LMC as separate study. Relevant metabolomics targets down-stream to predicted mutations will likely provide us with valid metabolomic targets as biomarkers.

Replacement of frequently false negative CSF cytology with more concrete CSF metabolomics for the diagnosis of LMC is a critical need. As we differentiate patients with LMC from other patients having high risk for developing LMC at 100% sensitivity, we expect to develop CSF metabolomic profiles as a screening test for LMC.l However, the usefulness as a screening method or for early diagnosis should not be overstated before prospective validation studies including false negative CSF samples with clinically or radiologically positive LMC cases and postoperative false positive CSF samples.

## MATERIALS AND METHODS

### Patient groups

We analyzed samples from five groups: 1) Group 1a, systemic cancer without CNS tumor; 2) Group 1b, no tumor; 3) Group 2, LMC; 4) Group 3, brain metastasis without LMC; 5) Group 4, brain tumor other than brain metastasis. To define Group 1a patients, recent images (≤ 3 months) such as computed tomography or positron-emission tomography scan or bone marrow reports were evaluated for existing cancer lesions. All Group 2 LMC patients had both a cytological diagnosis of LMC and positive neuroimaging study (gadolinium-enhanced brain MRI/whole-spine MRI) results [8]. To avoid including patients that had LMC in Group 3 or Group 4, those with less than 3 months follow-up after CSF sampling were excluded from the analysis.

### CSF archives

The CSF samples were obtained after Institutional Review Board (NCC-150002) approval; they were used to develop CSF biomarkers from patients who had already submitted informed consent. The CSF samples were obtained via lumbar puncture during spinal anesthesia (Group 1a) or CSF cytology examination (Group 2, Group 3, and Group 4). Other CSF samples were obtained from the cisternal/subarachnoid space during craniotomy (Group 1b, Group 3, and Group 4). The CSF sample was centrifuged (2,000 ´ g, 20 min) within 1 hour of collection, and then the supernatant was aliquoted. A 50-μl sample of each supernatant was used for MS analysis. The remaining samples were centrifuged again at 10,000 g for 30 min and kept frozen at –80°C for further study of genomic profiles and nano-sized particle evaluation.

### CSF extraction for metabolite profiling

The metabolites present in the CSF were extracted using a modified Bligh and Dyer method [13]. In brief, 50 μl CSF were added to 1 ml water. After vortexing, 2 ml MeOH and 0.9 ml dichloromethane were added. After vortexing and incubation on ice for 30 min, 1 ml water and 0.9 ml dichloromethane were added, then the mixture was centrifuged (1,000× g, 10 min, at room temperature). Nitrogen gas was used to dry the supernatant for MS analysis.

### CSF metabolite profiling using LC-MS/MS

The extracted metabolites were dried and then reconstituted in 0.1% formic acid and subjected to LC-MS/MS analysis. We used a Shimadzu Nexera X2 system (Shimadzu, Kyoto, Japan) coupled to a Sciex Triple TOF 5600+ system (Sciex, Framingham, MA, USA); the front end was equipped with a DuoSpray ion source. For the ultraLC separation, the sample was loaded into an Atlantis T3 sentry guard cartridge (3 μm, 2.1 × 10 mm; Waters, Milford, MA, USA). Separation was performed in an Atlantis T3 column (3 μm, 2.1 × 100 mm; Waters). The MS system was set to perform one full scan (50 to 1,200 *m/z* range) followed by LC-MS/MS of the 10 most abundant parent ions (mass tolerance, 50 mDa; collision energy, 35%). All data that indicated the presence of LMIs associated with metabolites were used for the LOMEs.

### LOMEs

### Candidate LMIs

PCA-DA was performed to distinguish Group 1b from a set of groups of 1a/2/3/4 and Group 2 from a set of groups of 1a/3/4. In both cases, each group was not split into training and validation sets because sample sizes were relatively small (especially for Group 3). PCA-DA requires an aligned peak table. Therefore, it is first necessary to import the LC/MS peak lists (.peaks file) into the MarkerView software (AB SCIEX, Foster City, CA). The parameters for this process were: retention time tolerance, 0.01 min; mass tolerance, 10.0 ppm; intensity threshold, 10; maximum number of peaks, 20000; and area reporting using the area integrated from raw data, not from the original peak finding. The consequent peak table was normalized using total area sums and transformed into common logarithms; Pareto-scaling was then performed. A mass intensity (peak area) of 0 was replaced with 1 because log_10_(0) is not defined and log_10_(1) becomes 0 again.

The PCA-DA DS was the weighted sum of the Pareto-scaled intensities of all detected LMIs (10,905 LMIs). However, only a small portion of the LMIs (i.e., candidate LMIs) mostly dominated the DSs. Search algorithm I was applied to the PCA-DA results to reveal a candidate set of *P* LMIs (Figure 5A) based on the following two requirements: 1) LMIs having weighted intensities of magnitude > 0.01 for each sample and 2) LMIs taken in common for more than half of the whole sample. The threshold of 0.01 was determined using a repetitive process of varying a threshold and then examining the number of the candidate LMIs and their separation performance. A peak table of only candidate LMIs was constructed and PCA-DA of the values in the reduced peak table was performed again to assign weighting factors for the candidate LMIs.

### Discriminative LMIs

The discriminative LMIs were determined from the *P* LMI candidates using search algorithm II (Figure 5). It consisted of germination, growth, and shrinkage modules. A seed set was created using the germination module and the growth and shrinkage modules were then interchangeably applied to the seed set until no more updates occurred. The last updated set was designated as the discriminative set of LMIs.

Germination module construction consisted of the following procedures: 1) whether there was a single LMI with a sensitivity and specificity of 100% was checked, 2) the sums of the sensitivity and specificity for _*P*_C_2_ and _*P*_C_3_ combinations were computed, 3) the set of two or three LMIs with the greatest value for the sum of sensitivity and specificity was set aside and step 2) was repeated using the remaining LMIs while the number of the remnants was ≥ 2, 4) any set of two or three LMIs was regarded as a single LMI and steps 2) – 3) were repeated, 5) Step 4) was repeated. The set combined at the preceding repetition was regarded as a single LMI at the subsequent repetition. 6) the set of *S* LMIs (Figure 5A) with the greatest value for the sum of sensitivity and specificity was designated as the seed set.

The following procedures the growth module construction; 7) the _*R*_C_1_, _*R*_C_2_, and _*R*_C_3_ combinations were added to the seed set, where *R* = *P* – *S,* 8) the enlarged seed set with the greatest value for the sum of sensitivity and specificity was designated as a new seed set if the enlarged seed set was better than the former seed set in view of the sum of sensitivity and specificity, and step 7) was repeated with the remaining LMIs, 9) the final renewed seed set was designated as the discriminative set of LMIs.

Finally, the shrinkage module included procedures similar to, but opposite from, construction of the growth module; 10) the _*R*_C_1_, _*R*_C_2_, and _*R*_C_3_ combinations were subtracted from the seed set, where *R* = *P* – *S*, 11) the reduced seed set with the greatest value for the sum of sensitivity and specificity was designated as a new seed set if the reduced seed set was better than the former seed set in view of the sum of sensitivity and specificity, and step 10) was repeated with the remaining LMIs, 12) The final renewed seed set was designated as the discriminative set of LMIs.

When the number of sets with the greatest value for the sum of sensitivity and specificity was > 1, one was chosen using the following two criteria: Priority 1) when the numbers of LMIs in the sets with the same value for the sum of sensitivity and specificity were different, the set with the fewest LMIs was chosen. Priority 2) when the numbers of LMIs in the sets were the same, the set with the maximum Fisher's discriminant ratio was chosen.

### Identification of metabolite ions

The MS and MS/MS spectra were submitted to the Formula Finder computational tools (Sciex) that proposes probable elemental compositions within a specified mass tolerance of a given mass-to-charge (*m/z*) ratio using PeakView software (Sciex). Using metabolite databases from the HMDB, specific compounds were found for the given *m/z*, listed in rank order based on the MS and MS/MS data. A proteomic MS/MS analysis was performed using Proteome Discoverer 1.4 software (Thermo Fisher Scientific, Waltham, MA, USA). MASCOT 2.3.2 and SEQUEST files were used for the search of the Uniprot database.

### Statistical analyses

Categorical data were analyzed using the chi-square, Fisher's exact, or Mann–Whitney *U*-tests as appropriate. Quantitative data were analyzed using one-way analysis of variance with post hoc comparisons (Scheffe's test). A two-tailed *p* value < 0.05 was considered to indicate a statistically significant result. All statistical analyses were performed using STATA 10.0 software (StataCorp LP, College Station, TX, USA).

## CONCLUSIONS

CSF metabolomic profiles differentiated five different cancer status groups. We found that these metabolic analyses could be used to diagnose LMC and exclude the high-risk group at accuracy of 100%. Future trials should reveal that this approach can also be used to differentiate false positive from proliferating LMC and also false negative due to rarity of floating cancer cells in CSF of patients with LMC. We expect that CSF metabolic profiles can support CSF cytology for the diagnosis of LMC or might be used as a screening method.

## SUPPLEMENTARY MATERIALS FIGURES AND TABLES





## Abbreviations

CNS: central nervous system
CSF: cerebrospinal fluid
LC: liquid chromatography
LMC: leptomeningeal carcinomatosis
LMIs: low-mass ions
MS: mass spectrometry
PCA-DA: principal component analysis-based discriminant analysis
