# Supplementary material for: Cerebrospinal fluid metabolomic profiles can discriminate patients with leptomeningeal carcinomatosis from patients at high risk for leptomeningeal metastasis
Source: Oncotarget. 2017 Sep 18;8(60):101203–14. doi: 10.18632/oncotarget.20983 (PMC5731867; doi:10.18632/oncotarget.20983)
Supplement: Supplementary file 1 [file oncotarget-08-101203-s001.pdf]

Cerebrospinal fluid metabolomic profiles can discriminate patients with leptomeningeal carcinomatosis from patients at high risk for leptomeningeal metastasis

SUPPLEMENTARY MATERIALS

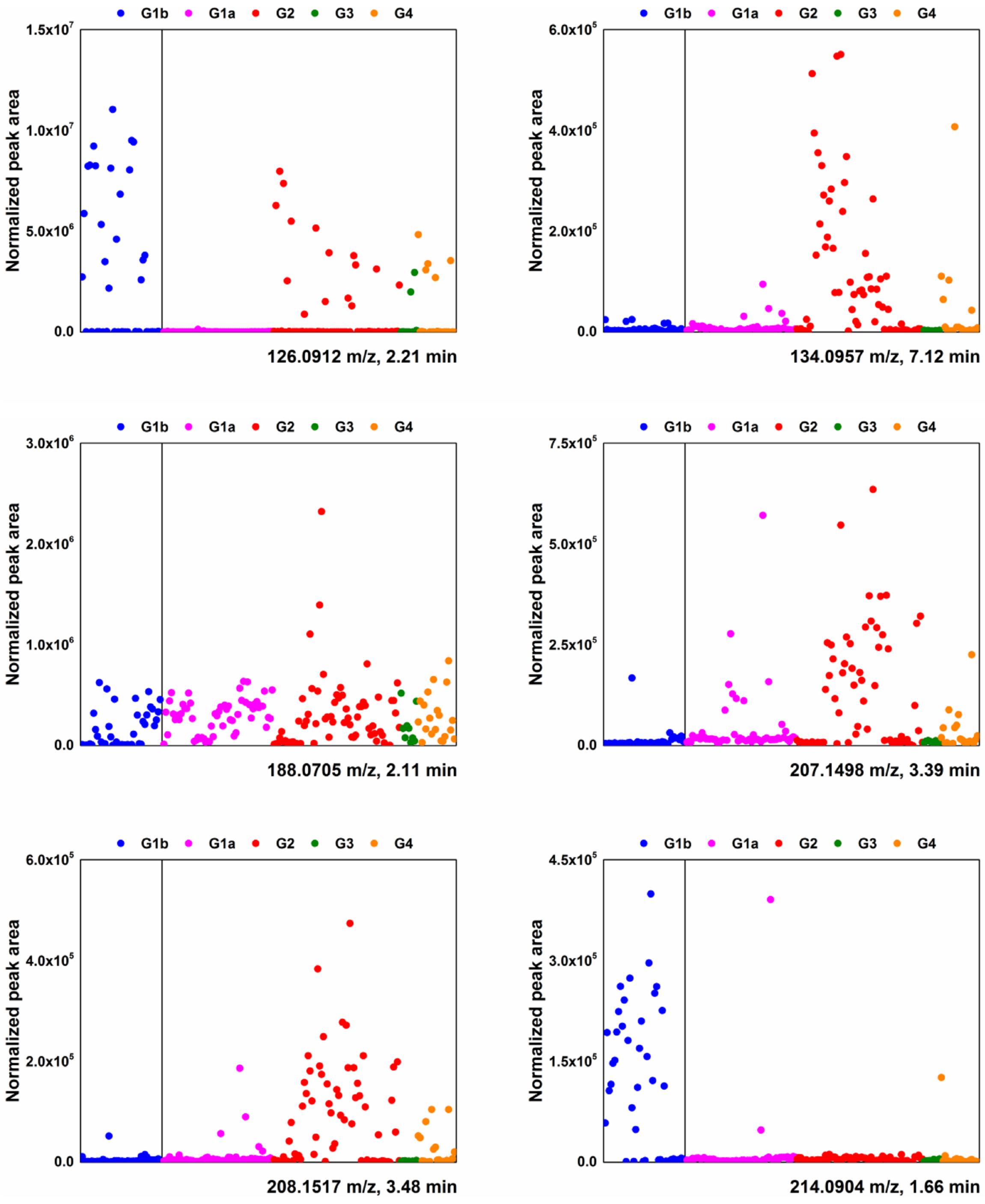

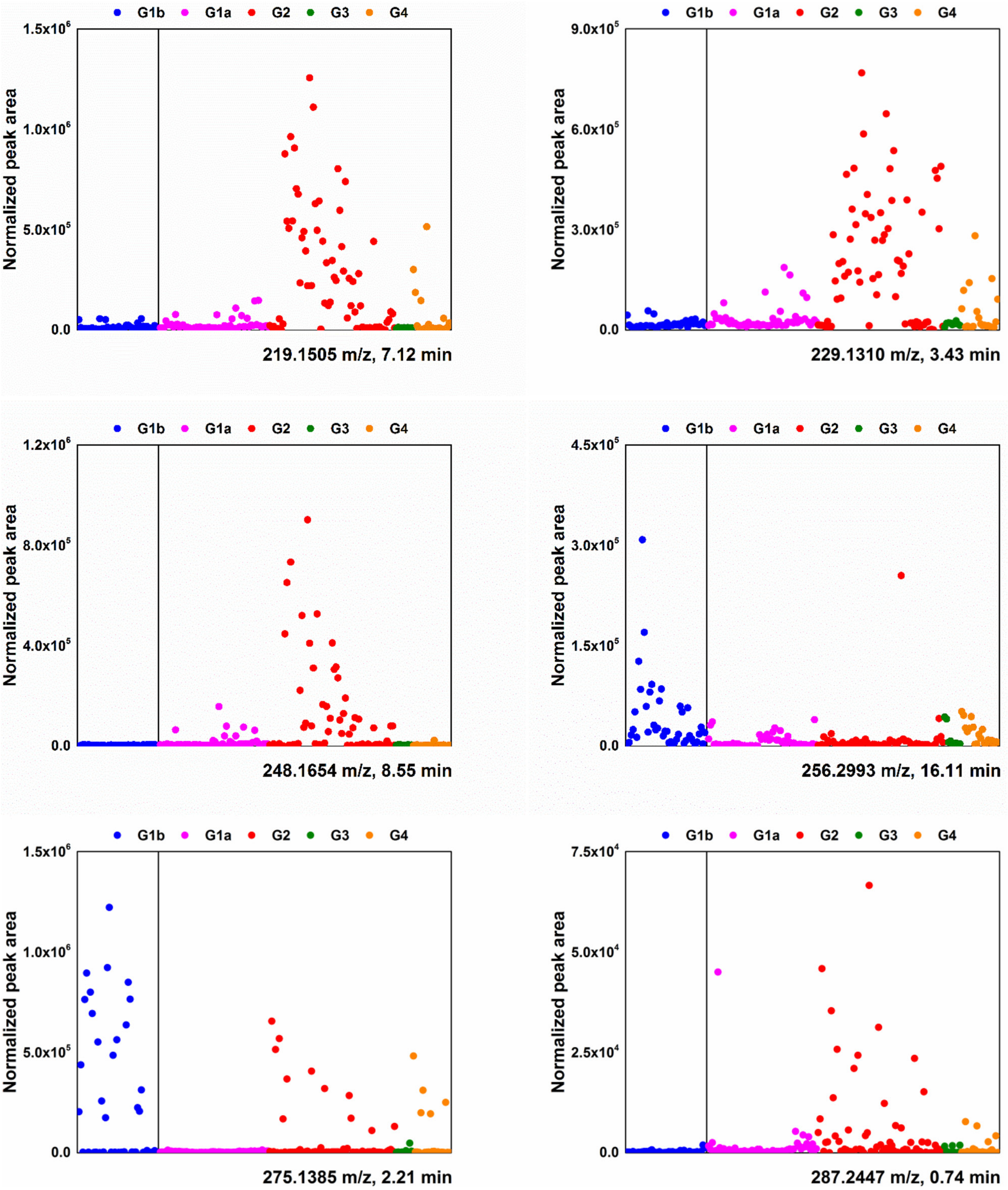
G1b G1a G2 G3 G4
Normalized peak area
1.5x10^6
1.0x10^6
5.0x10^5
0.0
219.1505 m/z, 7.12 min
9.0x10^5
6.0x10^5
3.0x10^5
229.1310 m/z, 3.43 min
1.2x10^6
8.0x10^5
4.0x10^5
248.1654 m/z, 8.55 min
4.5x10^5
3.0x10^5
1.5x10^5
256.2993 m/z, 16.11 min
275.1385 m/z, 2.21 min
7.5x10^4
5.0x10^4
2.5x10^4
287.2447 m/z, 0.74 min

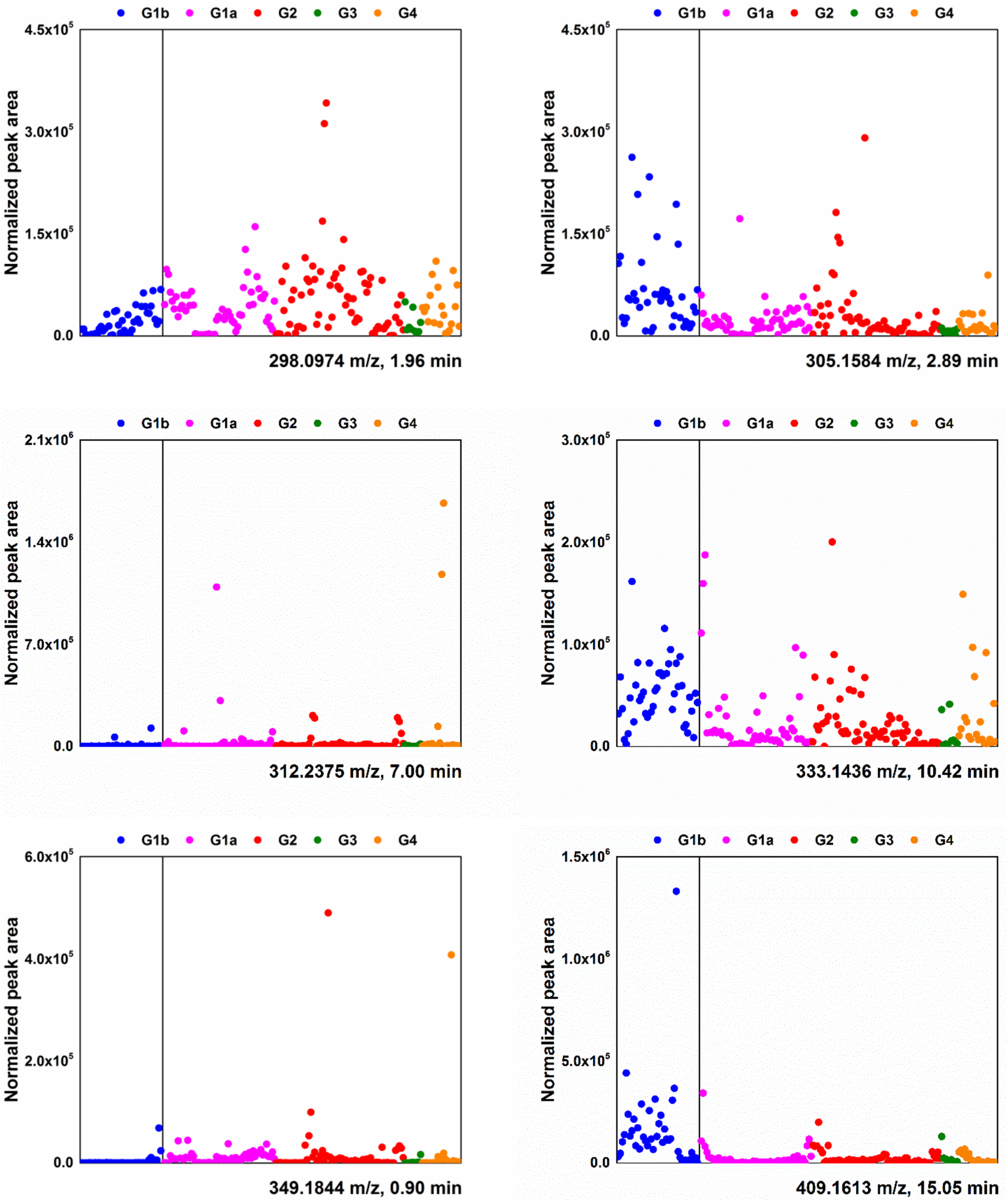

G1b
G1a
G2
G3
G4
Normalized peak area
4.5x10^5
3.0x10^5
1.5x10^5
0.0
298.0974 m/z, 1.96 min
305.1584 m/z, 2.89 min
2.1x10^6
1.4x10^6
7.0x10^5
312.2375 m/z, 7.00 min
3.0x10^5
2.0x10^5
1.0x10^5
333.1436 m/z, 10.42 min
6.0x10^5
4.0x10^5
2.0x10^5
349.1844 m/z, 0.90 min
1.5x10^6
1.0x10^6
5.0x10^5
409.1613 m/z, 15.05 min

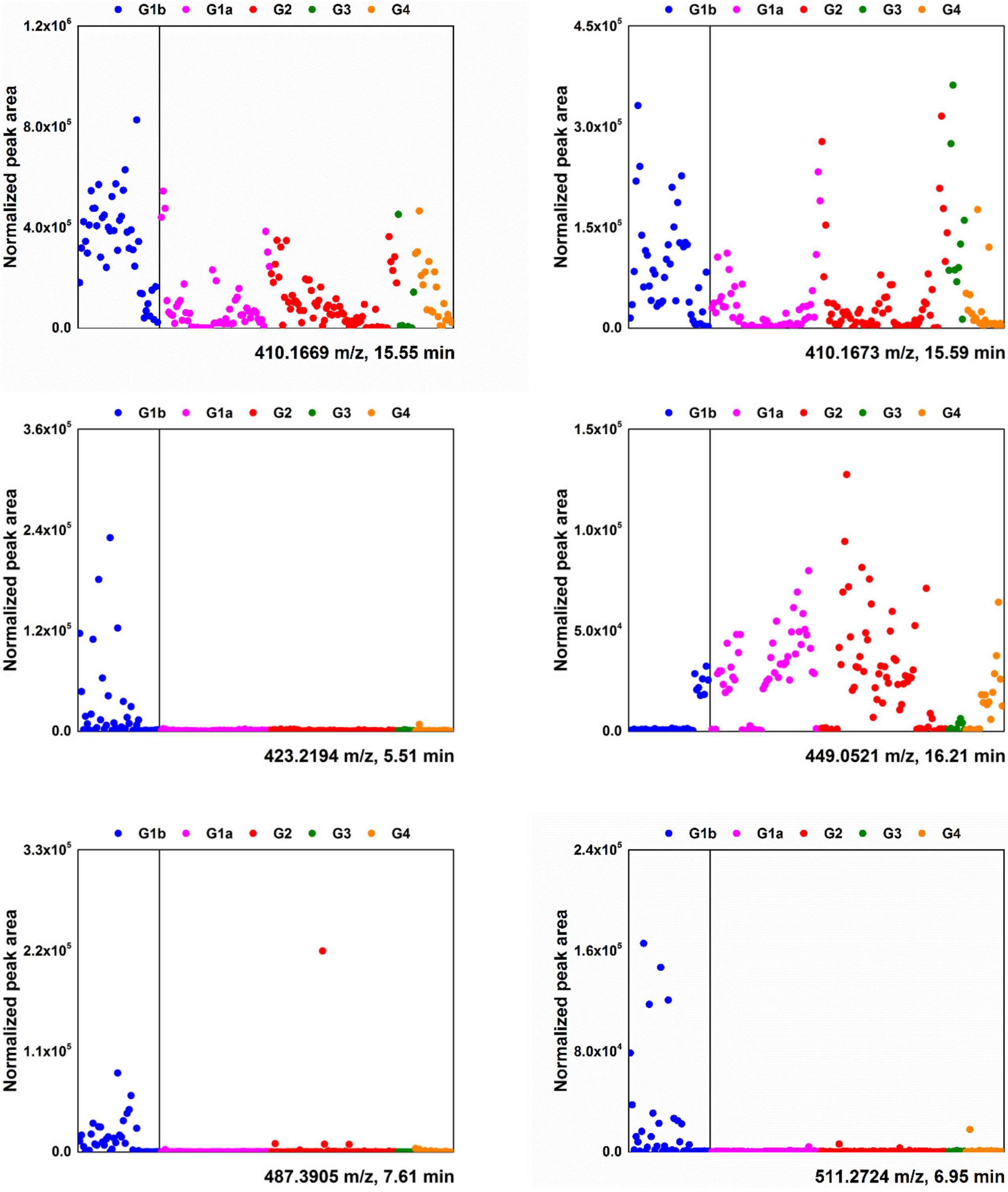
G1b G1a G2 G3 G4
Normalized peak area
1.2x10^6
8.0x10^5
4.0x10^5
0.0
410.1669 m/z, 15.55 min
4.5x10^5
3.0x10^5
1.5x10^5
0.0
410.1673 m/z, 15.59 min
3.6x10^5
2.4x10^5
1.2x10^5
0.0
423.2194 m/z, 5.51 min
1.5x10^5
1.0x10^5
5.0x10^4
0.0
449.0521 m/z, 16.21 min
3.3x10^5
2.2x10^5
1.1x10^5
0.0
487.3905 m/z, 7.61 min
2.4x10^5
1.6x10^5
8.0x10^4
0.0
511.2724 m/z, 6.95 min

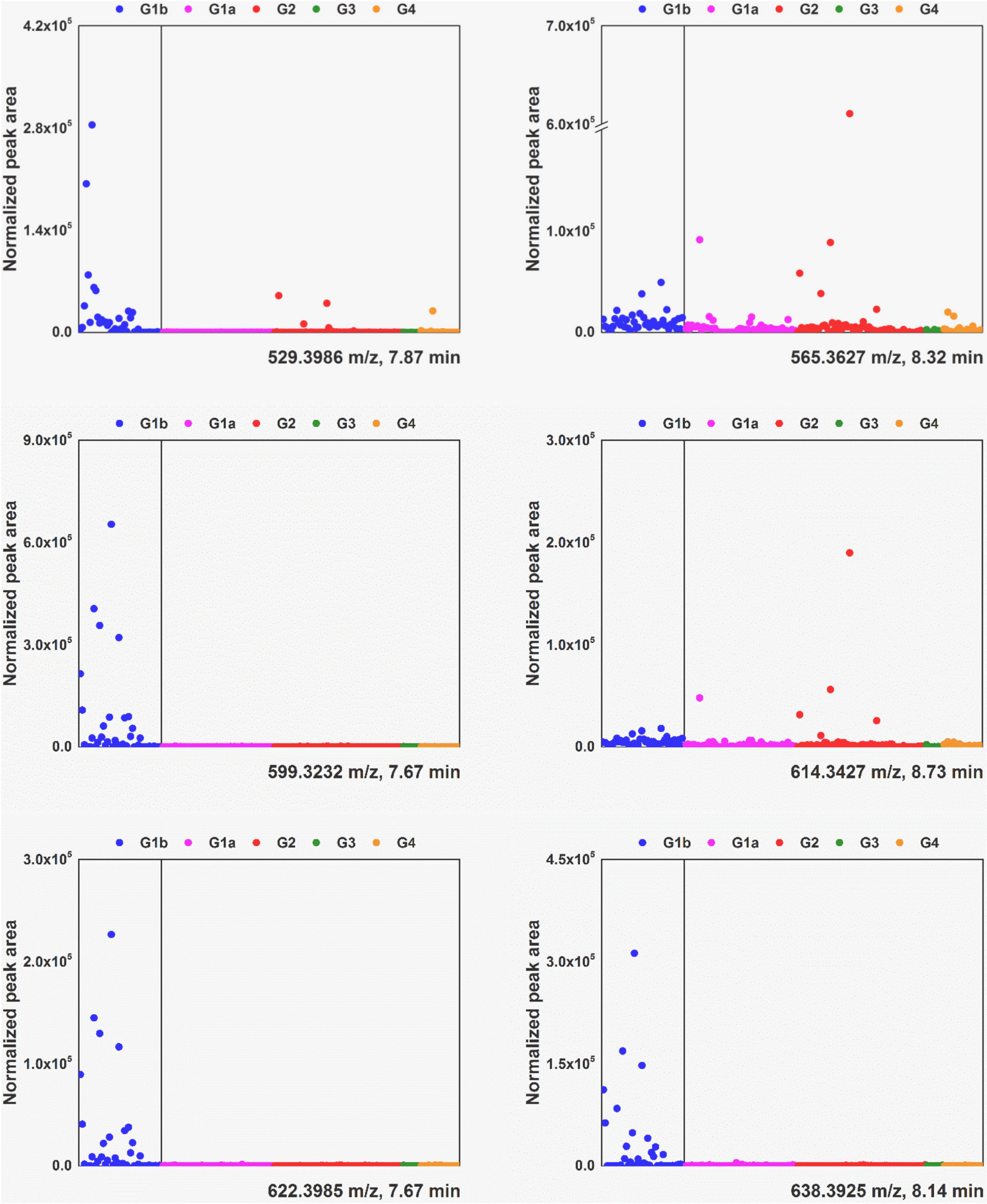
G1b G1a G2 G3 G4
Normalized peak area
4.2x10^5
2.8x10^5
1.4x10^5
0.0
529.3986 m/z, 7.87 min
7.0x10^5
6.0x10^5
1.0x10^5
0.0
565.3627 m/z, 8.32 min
9.0x10^5
6.0x10^5
3.0x10^5
0.0
599.3232 m/z, 7.67 min
3.0x10^5
2.0x10^5
1.0x10^5
0.0
614.3427 m/z, 8.73 min
3.0x10^5
2.0x10^5
1.0x10^5
0.0
622.3985 m/z, 7.67 min
4.5x10^5
3.0x10^5
1.5x10^5
0.0
638.3925 m/z, 8.14 min

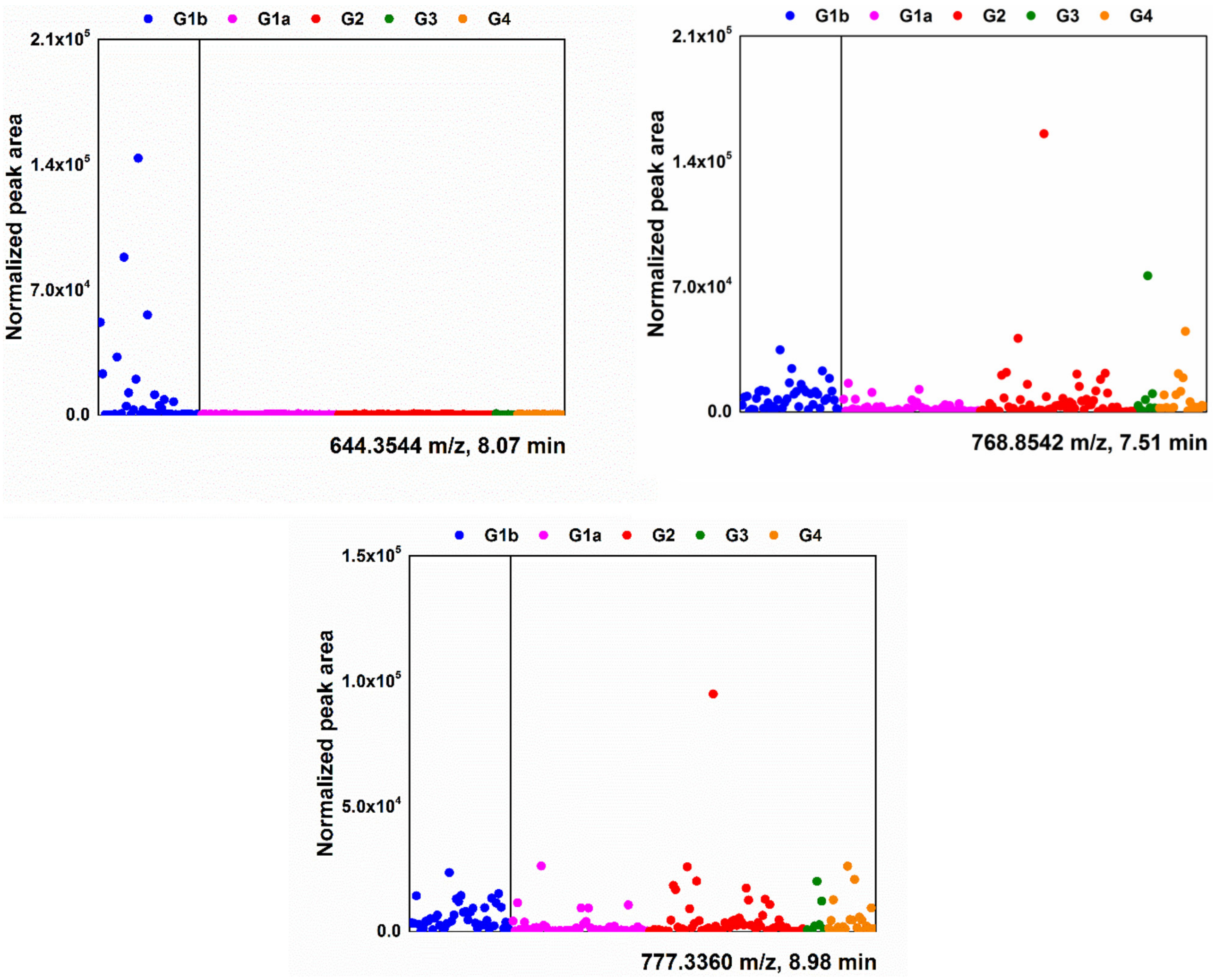

Supplementary Figure 1: Relative amounts of thirty-three low-mass ions discriminating cancer patients from patients without cancer. Normalized peak area (arbitrary unit) represents relative amounts of metabolites with a given *m/z*, in the CSF.

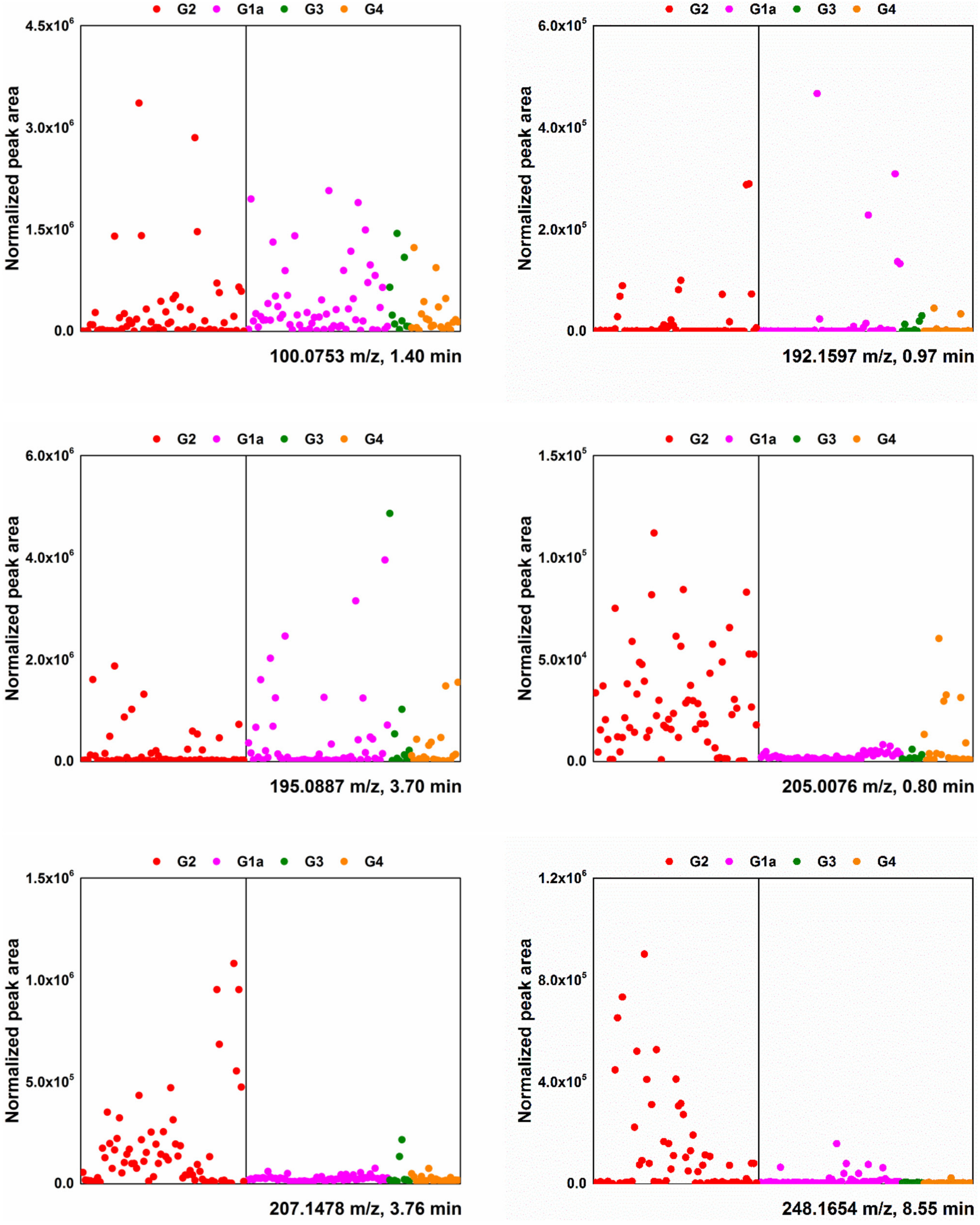
G2 G1a G3 G4
Normalized peak area
4.5x10^6
3.0x10^6
1.5x10^6
0.0
100.0753 m/z, 1.40 min
6.0x10^5
4.0x10^5
2.0x10^5
0.0
192.1597 m/z, 0.97 min
6.0x10^6
4.0x10^6
2.0x10^6
0.0
195.0887 m/z, 3.70 min
1.5x10^5
1.0x10^5
5.0x10^4
0.0
205.0076 m/z, 0.80 min
1.5x10^6
1.0x10^6
5.0x10^5
0.0
207.1478 m/z, 3.76 min
1.2x10^6
8.0x10^5
4.0x10^5
0.0
248.1654 m/z, 8.55 min

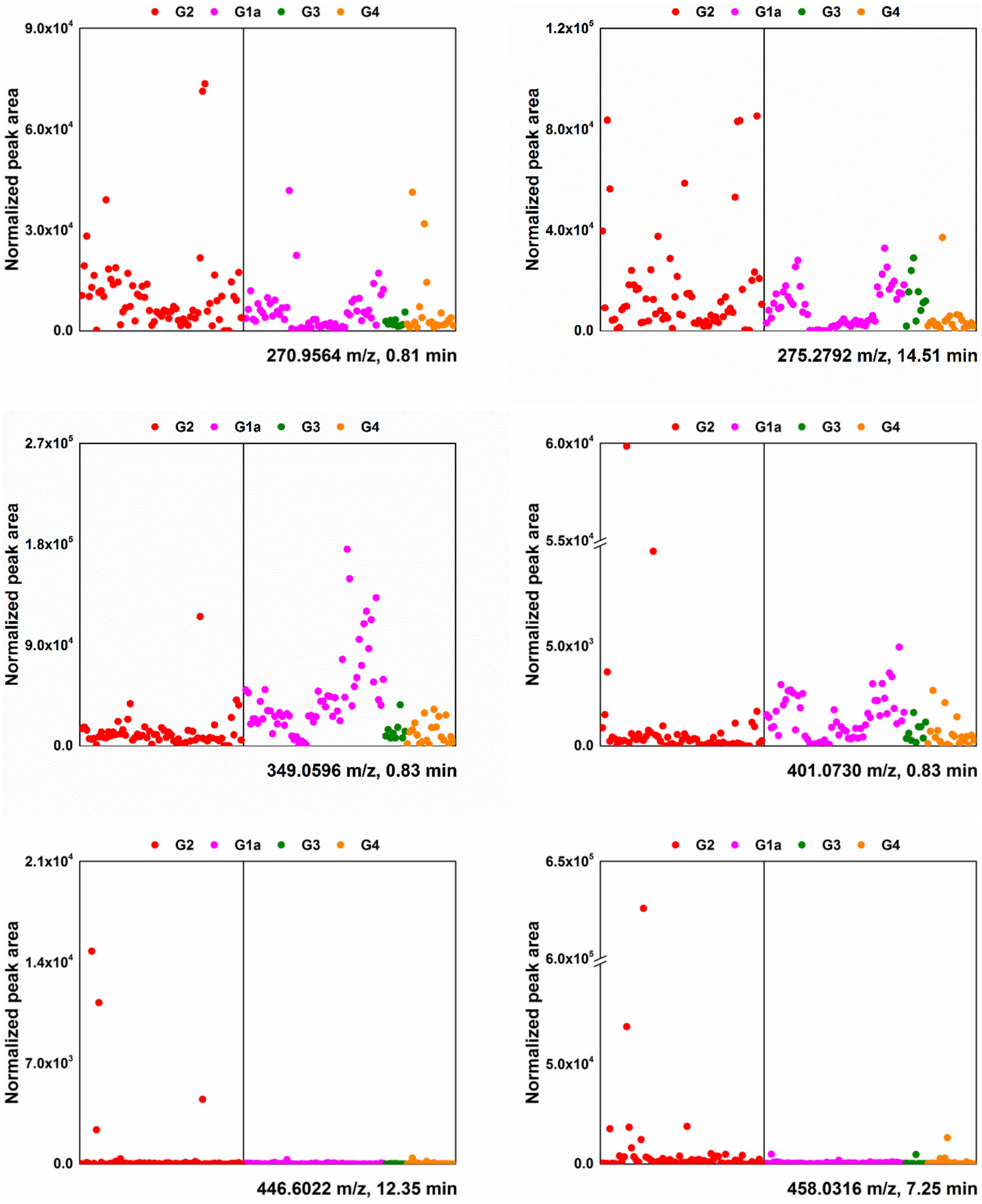
G2
G1a
G3
G4
Normalized peak area
9.0x10^4
6.0x10^4
3.0x10^4
0.0
270.9564 m/z, 0.81 min
1.2x10^5
8.0x10^4
4.0x10^4
0.0
275.2792 m/z, 14.51 min
2.7x10^5
1.8x10^5
9.0x10^4
0.0
349.0596 m/z, 0.83 min
6.0x10^4
5.5x10^4
5.0x10^3
0.0
401.0730 m/z, 0.83 min
2.1x10^4
1.4x10^4
7.0x10^3
0.0
446.6022 m/z, 12.35 min
6.5x10^5
6.0x10^5
5.0x10^4
0.0
458.0316 m/z, 7.25 min

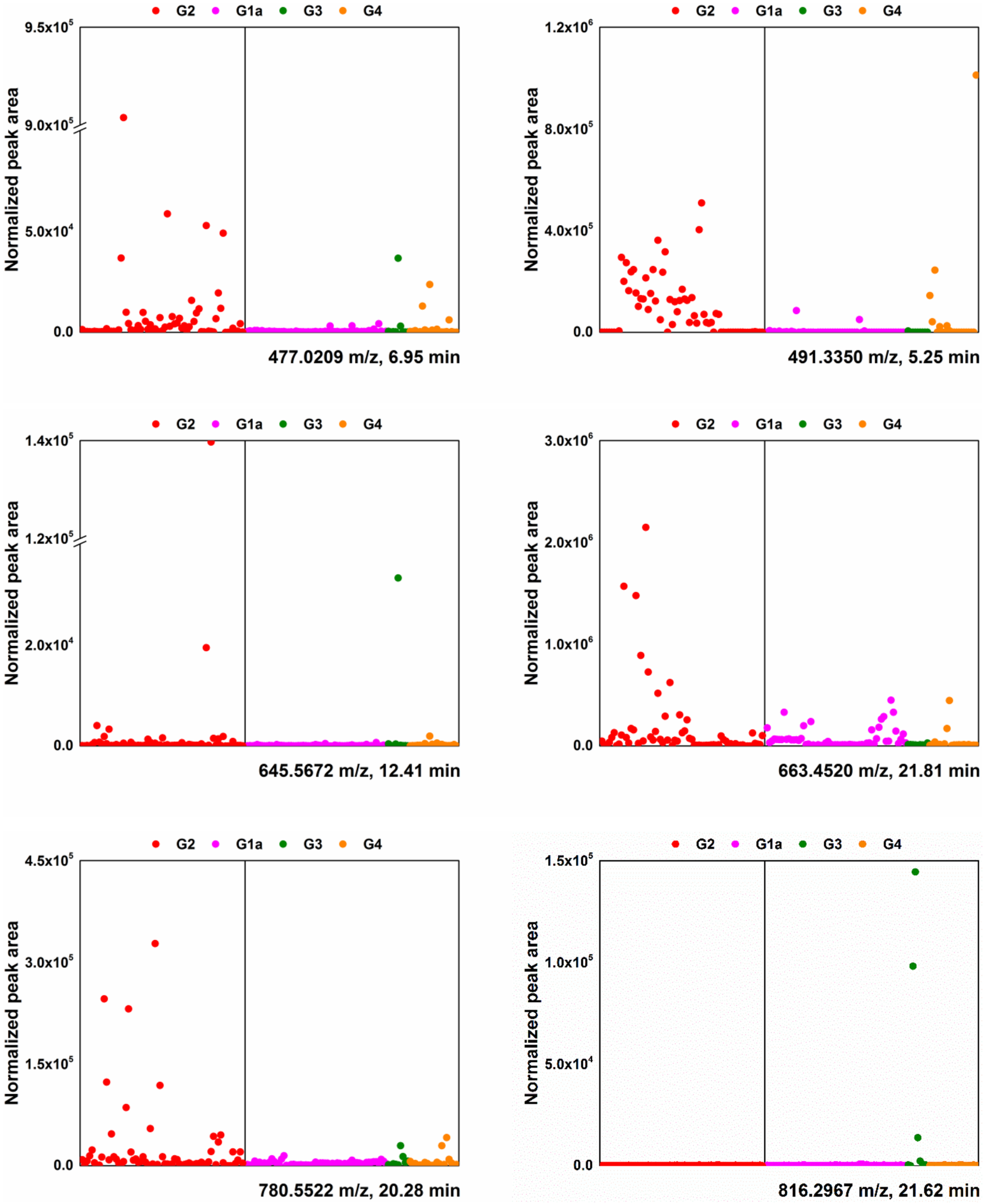

G2
G1a
G3
G4
Normalized peak area
9.5x10^5
9.0x10^5
5.0x10^4
0.0
477.0209 m/z, 6.95 min
1.2x10^6
8.0x10^5
4.0x10^5
0.0
491.3350 m/z, 5.25 min
1.4x10^5
1.2x10^5
2.0x10^4
0.0
645.5672 m/z, 12.41 min
3.0x10^6
2.0x10^6
1.0x10^6
0.0
663.4520 m/z, 21.81 min
4.5x10^5
3.0x10^5
1.5x10^5
0.0
780.5522 m/z, 20.28 min
1.5x10^5
1.0x10^5
5.0x10^4
0.0
816.2967 m/z, 21.62 min

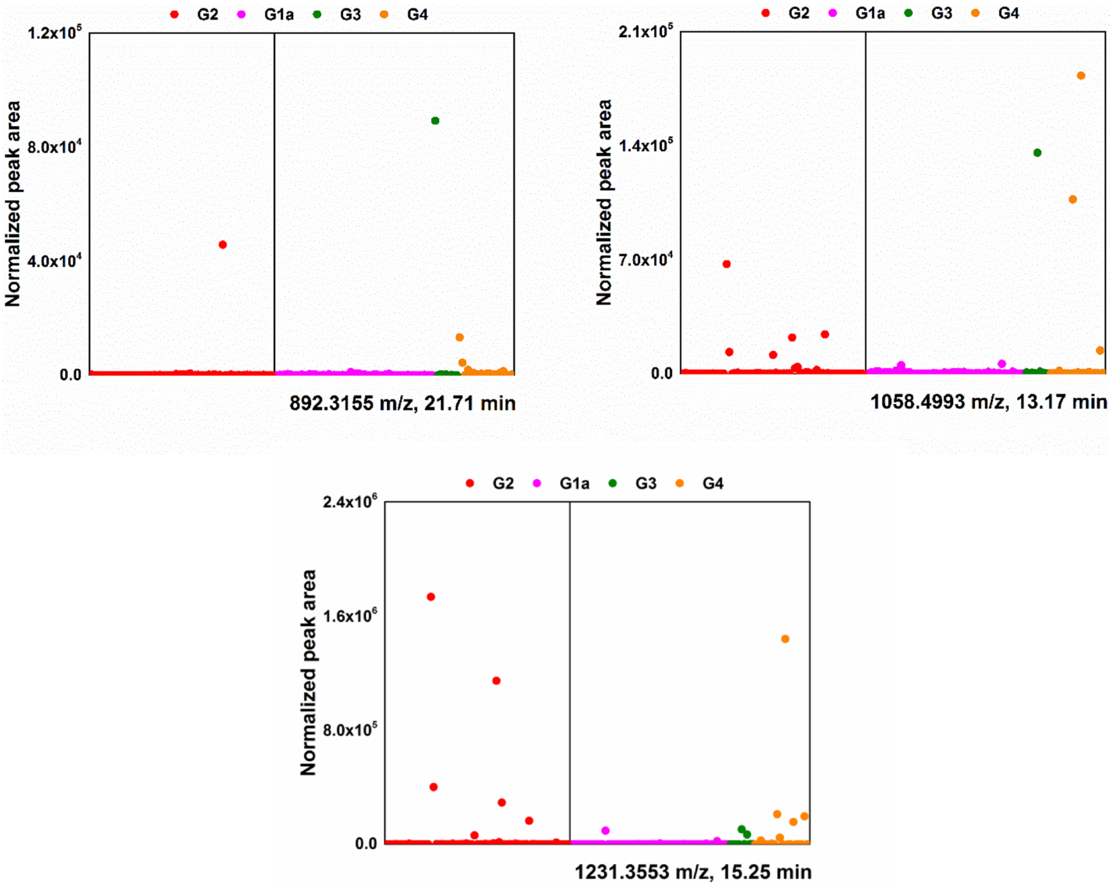

Supplementary Figure 2: Relative amounts of twenty-one low-mass ions discriminating patients with leptomeningeal metastasis from other groups of patients. Normalized peak area (arbitrary unit) represents relative amounts of metabolites with a given *m/z*, in the CSF.

Supplementary Table 1: Thirty-three low-mass ions discriminating cancer patients (groups of 1a, 2, 3 and 4) from patients without cancer (Group 1b). See_Supplementary_Table 1
