# Supplementary material for: Cerebrospinal fluid metabolomic profiles can discriminate patients with leptomeningeal carcinomatosis from patients at high risk for leptomeningeal metastasis
Source: Oncotarget. 2017 Sep 18;8(60):101203–14. doi: 10.18632/oncotarget.20983 (PMC5731867; doi:10.18632/oncotarget.20983)
Supplement: Supplementary file 2 [file oncotarget-08-101203-s002.docx]

Supplementary Table 1: Thirty-three low-mass ions discriminating cancer patients (groups of 1a, 2, 3 and 4) from patients without cancer (Group 1b).

| **Selected LMI (^a^*m/z*)** | **^b^Compound** | **Metabolite Name** | **Adduct** | **Adduct MW (Da)** | **Compound MW (Da)** | **Delta** |
| --- | --- | --- | --- | --- | --- | --- |
| 126.0912 | HMDB37853 | 2-Methyl-4-propyloxazole | M+H | 126.09134 | 125.084064 | 0.00014 |
|  | HMDB37854 | 4-Methyl-2-propyloxazole | M+H | 126.09134 | 125.084064 | 0.00014 |
|  | HMDB37861 | 5-Methyl-2-propyloxazole | M+H | 126.09134 | 125.084064 | 0.00014 |
|  | HMDB30345 | 6-Acetyl-1,2,3,4-tetrahydropyridine | M+H | 126.09134 | 125.084064 | 0.00014 |
|  | HMDB38288 | 4-Butyloxazole | M+H | 126.09134 | 125.084064 | 0.00014 |
|  | HMDB39583 | 2-Ethyl-5-imino-1-cyclopenten-1-ol | M+H | 126.09134 | 125.084064 | 0.00014 |
|  | HMDB34883 | 3,4-Dihydro-5-propanoyl-2H-pyrrole | M+H | 126.09134 | 125.084064 | 0.00014 |
|  | HMDB38289 | 5-Butyloxazole | M+H | 126.09134 | 125.084064 | 0.00014 |
|  | HMDB37866 | 4-Ethyl-2,5-dimethyloxazole | M+H | 126.09134 | 125.084064 | 0.00014 |
|  | HMDB37865 | 2-Ethyl-4,5-dimethyloxazole | M+H | 126.09134 | 125.084064 | 0.00014 |
|  | HMDB37867 | 5-Ethyl-2,4-dimethyloxazole | M+H | 126.09134 | 125.084064 | 0.00014 |
|  | HMDB37860 | 2-Methyl-5-propyloxazole | M+H | 126.09134 | 125.084064 | 0.00014 |
| 134.0957 | HMDB14890 | Tranylcypromine | M+H | 134.096425 | 133.0891494 | 0.000725 |
|  | HMDB12489 | 1,2,3,4-Tetrahydroisoquinoline | M+H | 134.096425 | 133.0891494 | 0.000725 |
| 188.0705 | HMDB00734 | Indoleacrylic acid | M+H | 188.070605 | 187.0633285 | 0.000105 |
| 207.1498 | HMDB41445 | Agrocybenine | M+H | 207.149189 | 206.1419132 | 0.000611 |
|  | HMDB60656 | Monoethylglycinexylidide | M+H | 207.149189 | 206.1419132 | 0.000611 |
| 208.1517 | HMDB30354 | Arenaine | M+H | 208.144438 | 207.1371622 | 0.007262 |
| 214.0904 | HMDB32418 | 2-(4-Methyl-5-thiazolyl)ethyl butanoate | M+H | 214.089625 | 213.0823494 | 0.000775 |
|  | HMDB32422 | 2-(4-Methyl-5-thiazolyl)ethyl isobutyrate | M+H | 214.089625 | 213.0823494 | 0.000775 |
| 219.1505 | HMDB02004 | 5-Methoxydimethyltryptamine | M+H | 219.149189 | 218.1419132 | 0.001311 |
|  | HMDB60625 | N-despropyl ropinirole | M+H | 219.149189 | 218.1419132 | 0.001311 |
| 229.1310 | HMDB60605 | Metyrapol | M+H | 229.133539 | 228.1262631 | 0.002539 |
| 248.1654 | HMDB14597 | Meperidine | M+H | 248.164505 | 247.1572289 | 0.000895 |
|  | HMDB41913 | Ketobemidone | M+H | 248.164505 | 247.1572289 | 0.000895 |
| 256.2993 | n.a. | | | | | |
| 275.1385 | n.a. | | | | | |
| 287.2447 | HMDB02172 | N1,N12-Diacetylspermine | M+H | 287.244152 | 286.2368762 | 0.000548 |
| 298.0974 | HMDB01173 | 5'-Methylthioadenosine | M+H | 298.096836 | 297.0895601 | 0.000564 |
| 305.1584 | n.a. | | | | | |
| 312.2375 | n.a. | | | | | |
| 333.1436 | HMDB60831 | meta-O-Dealkylated flecainide | M+H | 333.142053 | 332.1347771 | 0.001547 |
|  | HMDB14698 | Zanamivir | M+H | 333.140475 | 332.133199 | 0.003125 |
| 349.1844 | HMDB33011 | Foeniculoside IX | M+H | 349.185694 | 348.1784179 | 0.001294 |
|  | HMDB39014 | Nepetariaside | M+H | 349.185694 | 348.1784179 | 0.001294 |
|  | HMDB33219 | (1S,2S,4R,5S,7S)-2,5,7-Fenchanetriol 2-O-b-D-glucoside | M+H | 349.185694 | 348.1784179 | 0.001294 |
|  | HMDB34786 | (1S,2S,4S)-1,8-Epoxy-p-menthane-2,7-diol 2-O-b-D-glucoside | M+H | 349.185694 | 348.1784179 | 0.001294 |
|  | HMDB33009 | Foeniculoside VIII | M+H | 349.185694 | 348.1784179 | 0.001294 |
|  | HMDB34874 | Foeniculoside V | M+H | 349.185694 | 348.1784179 | 0.001294 |
|  | HMDB33645 | (1S,2S,4S,5S)-2,4,7-Thujanetriol 4-glucoside | M+H | 349.185694 | 348.1784179 | 0.001294 |
|  | HMDB41546 | Foeniculoside VII | M+H | 349.185694 | 348.1784179 | 0.001294 |
|  | HMDB33236 | cis-10-Hydroxylinalyl oxide 7-glucoside | M+H | 349.185694 | 348.1784179 | 0.001294 |
|  | HMDB39469 | (1S,2S,4R)-p-Menth-8-ene-1,2,10-triol 2-glucoside | M+H | 349.185694 | 348.1784179 | 0.001294 |
| 409.1613 | HMDB60830 | Melatonin glucuronide | M+H | 409.160542 | 408.1532658 | 0.000758 |
|  | HMDB37299 | 6,8-Dihydroxy-1,7-diprenylxanthone-2-carboxylic acid | M+H | 409.164565 | 408.1572885 | 0.003265 |
|  | HMDB36970 | Garcimangosone B | M+H | 409.164565 | 408.1572885 | 0.003265 |
|  | HMDB31920 | 9-Hydroxycalabaxanthone | M+H | 409.164565 | 408.1572885 | 0.003265 |
|  | HMDB37241 | 8,9-Dihydro-5-hydroxy-8-(1-hydroxy-1-methylethyl)-6-(2-methyl-1-oxopropyl)-4-phenyl-2H-furo[2,3-h]-1-benzopyran-2-one | M+H | 409.164565 | 408.1572885 | 0.003265 |
|  | HMDB37242 | Mammea A/AC cyclo F | M+H | 409.164565 | 408.1572885 | 0.003265 |
| 410.1669 / 410.1673 | HMDB11596 | Queuosine | M+H | 410.167024 | 409.1597481 | 0.000124 |
|  | HMDB59662 | Q | M+H | 410.167024 | 409.1597481 | 0.000124 |
|  | HMDB60454 | Chondroitin D-glucuronate | M+H | 410.165687 | 409.1584107 | 0.001213 |
|  | HMDB60470 | Dermatan L-iduronate | M+H | 410.165687 | 409.1584107 | 0.001213 |
| 423.2194 | HMDB38109 | 1-Methoxyficifolinol | M+H | 423.2166 | 422.2093241 | 0.0028 |
|  | HMDB35066 | 2',4,4'-Trihydroxy-6'-methoxy-3',5'-diprenylchalcone | M+H | 423.2166 | 422.2093241 | 0.0028 |
|  | HMDB40605 | Kanzonol J | M+H | 423.2166 | 422.2093241 | 0.0028 |
| 449.0521 | n.a. | | | | | |
| 487.3905 | n.a. | | | | | |
| 511.2724 | HMDB41047 | Physagulin A | M+H | 511.26903 | 510.2617536 | 0.00337 |
| 529.3986 | n.a. | | | | | |
| 565.3627 | n.a. | | | | | |
| 599.3232 | HMDB61693 | 1-Oleoylglycerophosphoinositol | M+H | 599.31909 | 598.3118136 | 0.00411 |
| 614.3427 | n.a. | | | | | |
| 622.3985 | n.a. | | | | | |
| 638.3925 | n.a. | | | | | |
| 644.3544 | n.a. | | | | | |
| 768.8542 | Fibrinogen alpha chain (see Fig. 6) | | | | | |
| 777.3360 | Fibrinogen beta chain (see Fig. 6) | | | | | |

a, mass-to-charge ratio; b, information used in searching candidate metabolite in Human Metabolome Database (HMDB, http://www.hmdb.ca). Search condition; Mass tolerance ± 0.005 and H^+^ adduct in positive mode
